# Treatment strategies for triple-negative primary breast cancer in older women: a systematic review

**DOI:** 10.1093/jncics/pkaf049

**Published:** 2025-05-15

**Authors:** Buraq Ahmed, Qutaiba Al-Khames Aga, Kwok-Leung Cheung, Jana de Boniface, Michael Gnant, Maria-Joao Cardoso, Emad Rakha, Thiraviyam Elumalai, Nadia Harbeck, Orit Kaidar-Person, Amit Agrawal

**Affiliations:** Medical School, University of Cambridge, Cambridge, United Kingdom; Breast Surgery, Cambridge University Hospitals, Cambridge, United Kingdom; School of Pharmacy, University of Waterloo, Waterloo, Canada; School of Medicine, University of Nottingham, Derby, United Kingdom; Department of Medical Epidemiology and Biostatistics, Karolinska Institutet, Stockholm, Sweden, and Department of Surgery, Capio St Göran’s Hospital, Stockholm, Sweden; Comprehensive Cancer Center, Medical University of Vienna, Vienna, Austria; Austrian Breast & Colorectal Cancer Study Group, Vienna, Austria; Breast Unit, Champalimaud Foundation and Lisbon University Faculty of Medicine, Lisbon, Portugal; Pathology Department, University of Nottingham, and Nottingham University Hospitals NHS Trust, Nottingham, United Kingdom; Breast Oncology, Cambridge University Hospitals, Cambridge, United Kingdom; Breast Center, Department of OB&GYN and CCCMunich, LMU University Hospital, Munich, Germany; Breast Radiation Unit, Sheba Tel Hashomer, Ramat Gan, Israel; School of Medicine, Faculty of Medical & Health Sciences, Tel Aviv University, Tel Aviv, Israel; Medical School, University of Cambridge, Cambridge, United Kingdom; Breast Surgery, Cambridge University Hospitals, Cambridge, United Kingdom

## Abstract

**Background:**

Although the relative proportion of triple-negative breast cancer decreases with age, its prevalence is rising with an aging population. This study examined real-world treatment practices, whether age in older women with triple-negative breast cancer affects therapy and outcomes, focusing on the potentially curable nature of early-stage triple-negative breast cancer.

**Methods:**

A Preferred Reporting Items for Systematic Reviews and Meta-Analyses, PRISMA–compliant search using population, intervention, comparison, outcomes criteria identified literature from 2014 to 2023 across 5 databases (MEDLINE, Embase, PubMed, Web of Science, and Scopus), focusing on women aged 65 years and older with early-stage triple-negative breast cancer.

**Results:**

From 7171 records, 37 studies were included. Older women with triple-negative breast cancer exhibited less aggressive features, including lower Ki67, higher androgen receptor, and higher Bcl2 expression. Breast-conserving surgery with radiation therapy (RT) was associated with improved overall survival and breast cancer–specific survival, with fewer recurrences compared with mastectomy with or without RT. Older women with triple-negative breast cancer were more likely to receive RT than systemic therapy, and the lack of RT correlated with worse outcomes. Multivariate analyses showed that systemic treatment improved 5-year overall survival and breast cancer–specific survival. Overall, outcomes did not show significant differences between women aged 70 years and older and women younger than 70 years at a median follow-up of 46 months.

**Conclusions:**

The lack of overall outcome improvements for older women with triple-negative breast cancer following treatment may not solely be due to absent targetable receptors because the intrinsic biology in older patients may be relatively favorable. Instead, treatment selection biases against active treatment due to age-related factors may contribute substantially. Treatment decisions should be biology based and guided by a multidisciplinary, holistic, and patient-centered approach that carefully considers comorbidities, functional status, social support, and patient preferences.

## Introduction

Globally, more than 30% of breast cancer cases occur in women older than 70 years of age.^1^ Triple-negative breast cancer, a heterogeneous subtype characterized by the absence of estrogen, progesterone, and ERBB2 (formerly HER2) receptor expression, accounts for approximately 15% of all breast cancer cases.^2^ Although the relative proportion of triple-negative breast cancer decreases with age,^3^ there is an increasing prevalence of older women with triple-negative breast cancer due to the aging population.^4^

Triple-negative breast cancer is often linked to a poorer prognosis and is more prevalent among African American women, younger individuals, and tumors of higher grade and stage.^5^^,^^6^ The most common histological subtype is infiltrating ductal carcinoma of no special type, followed by metaplastic and pleomorphic invasive lobular carcinoma. Rare subtypes, accounting for less than 2% of cases, are generally well-differentiated, low-grade tumors and are more frequently observed in older patients compared with the no special type category. These rarer triple-negative breast cancer subtypes include low-grade adenosquamous carcinoma, fibromatosis-like spindle cell carcinoma, salivary gland–like tumors (adenoid cystic and mucoepidermoid carcinoma), and apocrine and secretory carcinoma.

Distant recurrence in triple-negative breast cancer peaks approximately 3 years after diagnosis and often involves visceral organs.^7^ In addition, 11.5% to 14% of de novo metastatic triple-negative breast cancer cases present with brain metastases.^8^ Distant recurrences substantially reduce survival, necessitating more aggressive and expedited treatment approaches, including for older women, because these recurrences directly affect their lifespan.^9^^,^^10^

The standard of care for early breast cancer includes local control through breast-conserving surgery with postoperative radiation therapy (RT) or mastectomy with or without RT, combined with or without systemic therapy. For triple-negative breast cancer, primary systemic therapy (or neo-adjuvant systemic therapy) is recommended for T2N0 or any TN1+ disease and above.^11^ This therapy typically involves cytotoxic regimens, such as sequential anthracycline-based and taxane-based systemic therapy.^12^^,^^13^ Recent evidence, however, supports the integration of platinum-based agents in the primary systemic therapy setting, particularly to enhance pathologic complete response rates. Trials such as CALGB 40603 (Alliance) and GeparSixto demonstrated improved pathologic complete response rates by adding carboplatin to triple-negative breast cancer regimes. This approach is increasingly adopted in clinical practice for appropriately selected older patients.^14^^,^^15^ Recently, immune checkpoint inhibitors, such as pembrolizumab, have been introduced for primary systemic and adjuvant settings.^16^^,^^17^ Poly(ADP-ribose) polymerase (PARP) inhibitors such as olaparib have shown efficacy in the primary systemic therapy or adjuvant setting for patients with germline *BRCA* mutations. These agents, however, are not approved for routine use in the primary systemic therapy setting.^18^

Systemic therapy can lead to toxicity that may be less tolerable in older women, substantially impairing their quality of life (QOL)^19^ due to comorbidities, polypharmacy, and frailty. The use of primary systemic therapy and breast-conserving surgery for triple-negative breast cancer decreases with age, even after accounting for cancer stage and comorbidities.^20^ Appropriate use of therapies may be influenced by clinicians’ erroneous subjective frailty assessments, highlighting the need for objective frailty measurement tools. Integrating these tools with other relevant factors ensures that treatment decisions prioritize the disease’s biological characteristics.^21^^,^^22^

The literature on treatment strategies and outcomes in older women with triple-negative breast cancer is limited, partly because of a lack of population-based data. Older patients are often excluded from randomized controlled trials due to age restrictions, making robust data collection difficult. Future randomized controlled trials involving this population are unlikely in the near term.^23^

This systematic review aims to synthesize real-world treatment patterns and outcomes in older women with early-stage triple-negative breast cancer. The review explores differences in treatment receipt and survival outcomes across 3 key dimensions: age (older vs younger patients), treatment intensity (treated vs untreated), and treatment type.

## Materials and methods

### Selection criteria

The inclusion and exclusion criteria were developed using the population, intervention, comparison, outcomes framework:

- **Population (P):** Women with triple-negative breast cancer as the subtype 70 years of age or older, per the International Society of Geriatric Oncology (SIOG) definition. Given the variability in defining *older,* studies defining this population as 65 years of age and older were also included.
- **Intervention (I):** Patients who underwent or were given the option of surgery, postoperative RT, or systemic therapy.
- **Comparison (C):** Other therapeutic options, younger patients, or no treatment.
- **Outcomes (O):** Any metric assessing 1 or more outcomes, including cancer-specific outcomes (disease-free survival [DFS] or recurrence), toxicity, or survival (breast cancer specific or overall).

The search was limited to English-language studies. Exclusion criteria included case reports, studies involving male patients (due to limited literature), and papers on preidentified genetic abnormalities.

### Search strategy

**Figure 1.**
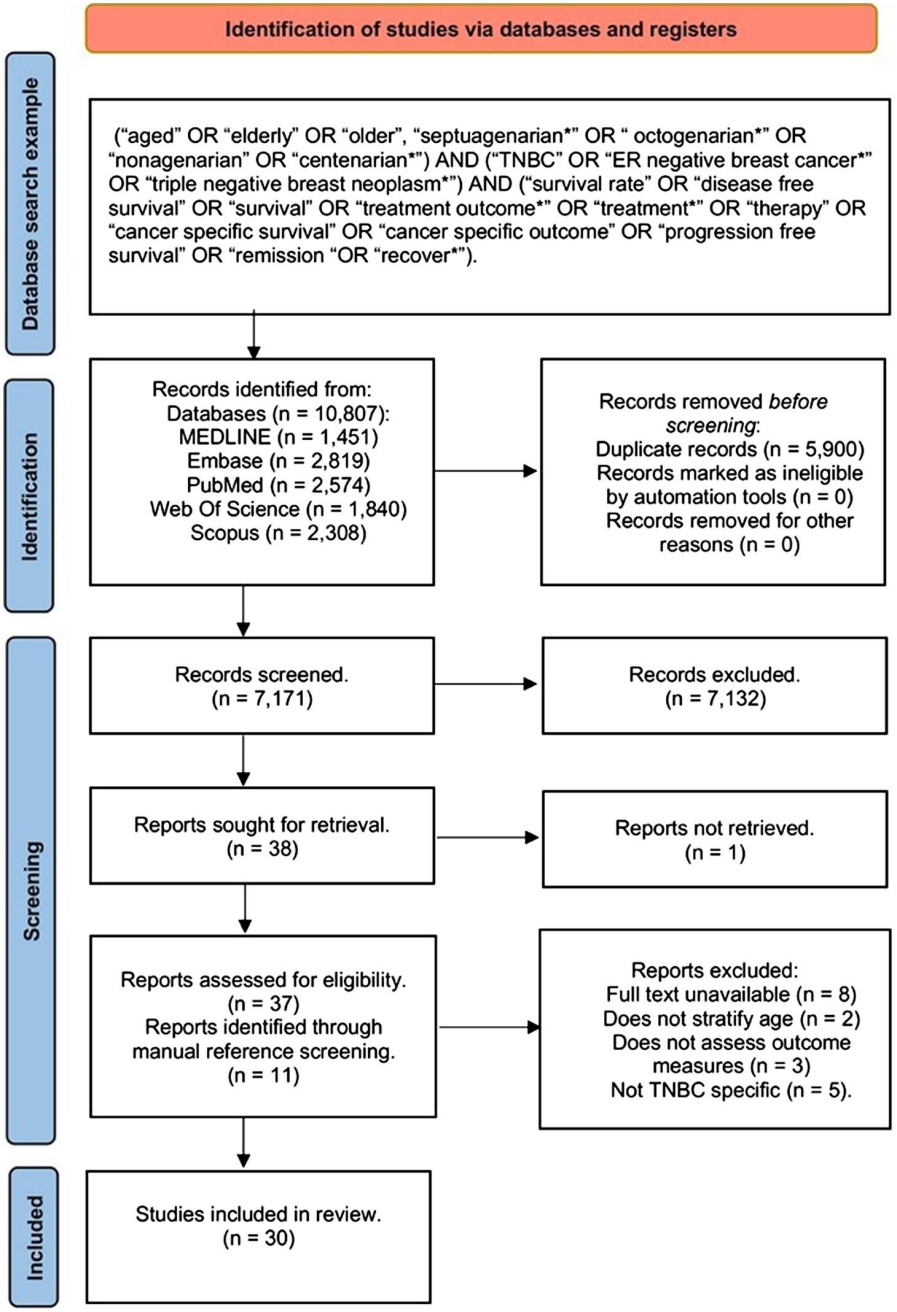
Preferred Reporting Items for Systematic Reviews and Meta-Analyses flow chart.

This review adhered to the Preferred Reporting Items for Systematic Reviews and Meta-Analyses (PRISMA) guidelines.^24^ Medical Subject Headings (MeSH) terms and free-text keywords (Supplementary Materials A) were used to search MEDLINE, Embase, PubMed, Web of Science, and Scopus. We searched these 5 databases (Figure 1) for publications from the last decade (2014-2023) to align with recent advancements in treating patients regardless of age and updated treatment guidelines. In addition, we manually searched reference lists of papers identified during abstract screening for relevant studies.

B.A. and A.A. created the search strategy and inclusion criteria. Two reviewers (B.A. and Q.A.A.) independently screened studies for eligibility and extracted data. Any discrepancies were resolved through discussion between the reviewers. If no consensus was reached, a third independent reviewer (A.A.) was consulted.

### Data collection

The extracted data included study identifiers (authors, year, country, study type, data source), population characteristics (age range, number of patients), tumor features (stage, grade, receptor status), treatment modalities evaluated (surgery, RT, systemic therapy, etc), and outcome measures (overall survival, breast cancer–specific survival, DFS, recurrence, toxicity). We also recorded author-reported study limitations as part of quality appraisal. Data extraction was performed independently by 2 reviewers (B.A. and Q.A.A.), and discrepancies were resolved through discussion or input from a third reviewer (A.A.) when needed.

### Quality assessment

The quality of the included primary studies was assessed using the Risk of Bias in Non-Randomised Studies of Interventions (ROBINS-I) tool.^25^

## Results

Our search, conducted in December 2023, identified 10 807 records, with 7171 remaining after duplicates were removed. Following Preferred Reporting Items for Systematic Reviews and Meta-Analyses screening guidelines, the title and abstract review reduced this number to 37 reports that met the inclusion criteria. However, 8 reports were excluded because only the abstracts were published. Reasons for exclusion during full-text screening included insufficient specificity for triple-negative breast cancer or inadequate age stratification (eg, defining *older* as ≥50 years). The search did not yield randomized controlled trials; most included studies were retrospective cohort studies conducted in the United States, Asia, and the United Kingdom.

### Quality assessment

Risk of bias was assessed using the ROBINS-I tool.^25^ Most studies were retrospective and rated as having a moderate overall risk of bias. Common concerns included confounding and selection bias, mainly due to unmeasured factors such as frailty and comorbidities.

Bias related to intervention classification, missing data, and outcome measurement was generally low, though a few studies showed moderate concerns in these domains. Selective reporting was rated moderate in most studies. A full breakdown of bias ratings across domains is provided in Supplementary Materials B.

### Surgery

The surgical literature on triple-negative breast cancer compares breast-conserving surgery plus RT and mastectomy with or without RT across various age groups. Three studies addressed this topic (Table 1; Table SB1): 2 based on the US Surveillance, Epidemiology, and End Results (SEER) Program cohort studies^26^^,^^27^ and 1 from an institutional database in China.^28^

**Table 1.** Papers primarily focused on locoregional interventions in older women with triple-negative breast cancer.

Author (year)	Study type	Patients meeting our study criteria, No. (age, y)	Treatment regimen investigated	Findings	Investigators’ recommended outcomes
Mburu et al., 2022^26^	Retrospective cohort study plus inverse probability treatment weighting analysis	4598 (>66)	Breast-conserving surgery; breast-conserving surgery plus RT; mastectomy; and mastectomy plus RT	Older women have higher death hazard Breast-conserving surgery plus RT lowers death incidence compared with breast-conserving surgery alone after multivariate analysis (*P < .*0001) 5-y cumulative death incidences: breast-conserving surgery plus RT (8.35%), mastectomy (18.56%), mastectomy plus RT (36.02%), breast-conserving surgery (20.44%) Breast-conserving surgery plus RT has lower death hazard Systemic therapy adjustment does not change findings Breast-conserving surgery plus RT improves overall survival, breast cancer–specific survival (stages I-II)	Omission of RT after breast-conserving surgery is problematic and needs urgent attention Breast-conserving surgery plus RT is associated with better prognosis in early-stage triple-negative breast cancer
Saifi et al. (2022)^27^	Retrospective cohort study plus propensity score matching	5743 (>60)	Breast-conserving surgery vs mastectomy	Breast-conserving surgery plus RT improves 5-y overall survival, breast cancer–specific survival (*P < .*001) Patients aged >60 y: better survival with breast-conserving surgery plus RT In patients who received systemic therapy, breast-conserving surgery plus RT has a higher 5-y overall survival (*P = .*007) and breast cancer–specific survival (*P = .*021) than patients undergoing mastectomy	Breast-conserving surgery plus RT is associated with better overall survival and breast cancer–specific survival compared with mastectomy in patients with triple-negative breast cancer
Zhong et al. (2020)^28^	Retrospective cohort study	450 (≥70)	Breast-conserving surgery without axillary lymph node dissection, sentinel lymph node biopsy, or RT	Breast-conserving surgery alone and mastectomy plus axillary lymph node dissection have similar 5-y DFS No overall survival or breast cancer–specific survival difference in triple-negative breast cancer	Breast-conserving surgery is suitable for older women with triple-negative breast cancer. Prognosis may depend on tumor characteristic

Abbreviations: DFS = disease-free survival; RT = radiation therapy.

The SEER study by Mburu et al.^26^ used inverse probability treatment weighting to investigate outcomes in older women with triple-negative breast cancer older than 66 years of age. Their analysis revealed the lowest cumulative incidence of death in patients undergoing breast-conserving surgery plus RT, particularly among women older than 75 years of age. These findings persisted after multivariate analysis and adjustments for systemic therapy. Improved overall survival and breast cancer–specific survival were observed in patients with stage III tumors undergoing breast-conserving surgery plus RT.^26^

The second SEER study, which employed propensity matching and focused on older women, also demonstrated improved 5-year overall survival and breast cancer–specific survival for patients undergoing breast-conserving surgery plus RT compared with patients treated with mastectomy alone.^27^ It is important to note that both SEER studies, with a combined sample size of 18 000, covered overlapping periods, raising potential concerns about data duplication.

In contrast, the study by Zhong et al.^28^ compared mastectomy with axillary lymph node dissection with breast-conserving surgery without axillary lymph node dissection, sentinel lymph node biopsy, or RT. They observed no statistically significant differences in DFS between these surgical approaches. The study noted higher rates of ipsilateral breast cancer recurrence in patients treated with breast-conserving surgery without RT, however, particularly for triple-negative breast cancer, compared with luminal subtypes. Notably, this study did not exclusively focus on triple-negative breast cancer and did not adjust for the impact of systemic therapy, indicating that some patients undergoing breast-conserving surgery may have also received systemic therapy. These findings align with the observation by Mburu et al.^26^ that survival rates were lower in patients undergoing breast-conserving surgery without RT than in patients undergoing mastectomy alone, potentially due to increased local recurrence rates.

Importantly, these observations do not necessarily extend to younger age groups. In a cohort of patients younger than 60 years of age, similar outcomes in terms of breast cancer–specific survival and overall survival were observed between breast-conserving surgery and mastectomy.^27^

### Postoperative RT

Eight retrospective cohort studies evaluated the role of postoperative RT in older women with triple-negative breast cancer (Table 2; Table SB2). These studies included work based on SEER^4^^,^^29^^,^^30^ and the US National Cancer Database^31^^,^^32^ registries and looked at outcomes following breast-conserving surgery, mastectomy, or both. Two pairs of studies used identical study periods (2004-2015), and another study used data from hospitals in Hong Kong, Malaysia, and Singapore between 2006 and 2012.^33^

**Table 2.** Papers primarily focused on postoperative RT in older women with triple-negative breast cancer.

Author (year)	Study type	Patients meeting our study criteria, No. (age, y)	Treatment regimen investigated	Findings	Investigators’ recommended outcomes
Xu et al. (2022)^29^	Retrospective cohort study with propensity score matching and nomogram	4465 (≥ 65)	RT after breast-conserving surgery	Older women with triple-negative breast cancer favor RT over systemic therapy ≥70 y: poorer overall survival, breast cancer–specific survival (*P < .*001) RT improves overall survival, breast cancer–specific survival in women ≥70 y	RT benefits older women with triple-negative breast cancer, except low-risk subgroup (identified through nomogram); high-risk group requires RT
Tang et al. (2022)^4^	Retrospective cohort study plus nomogram	4761 (≥70)	Effects of adjuvant systemic therapy or postoperative RT on overall survival and breast cancer–specific survival	RT improves overall survival and breast cancer–specific survival (*P < .*0001) No chemoradiation therapy linked to worst survival (*P < .*0001) Age, systemic therapy, RT predict overall survival (*p* < .001)	Results support RT in older women with triple-negative breast cancer
Algan et al. (2016)^36^	Retrospective cohort study	10 079 (≥70)	Impact of postoperative RT in triple-negative breast cancer	RT improves triple-negative breast cancer overall survival significantly RT use decreases with increasing age	There is improvement in overall survival with the addition of RT after breast-conserving surgery
Haque et al. (2019)^31^	Retrospective cohort study plus propensity score matching	8526 (≥70)	Omission of radiation post breast-conserving surgery in older women with triple-negative breast cancer	RT use decreases with age Adjuvant RT improves 5-y overall survival (*P < .*001) Omission of RT linked to poorer overall survival (*P < .*001) No RT cohort has higher mastectomy rate, diminishes with age	Caution must be exercised when considering omission of adjuvant RT in patients with triple-negative breast cancer patients
Haque et al. (2017)^37^	Retrospective cohort study	788 (≥70)	Postoperative RT utilization in older patients with breast cancers	Adjuvant RT improves breast cancer–specific survival in estrogen receptor-negative/ERBB2-negative breast cancer Poor differentiation, estrogen receptor-negative/ERBB2-negative breast cancer favors RT; predicts poorer outcomes	Age should not be a consideration to change management in triple-negative breast cancer
Crozier et al. (2020)^32^	Retrospective cohort study plus propensity score matching	16 062 (≥70)	Addition of systemic therapy to local therapy on overall survival in older women with triple-negative breast cancer	Systemic therapy improves overall survival (*P < .*0001), persisting after adjustment for node status and comorbidity RT improves overall survival (*P < .*0001)	Data support considering systemic therapy and RT in treating women aged ≥70 y with triple-negative breast cancer
Bhoo-Pathy et al. (2014)^33^	Retrospective cohort study	1138 (≥65)	Comparing survival between patients receiving mastectomy only, breast-conserving surgery, and mastectomy with RT	Age modifies locoregional treatment impact on survival Older age: No survival advantage with breast-conserving surgery with RT, postmastectomy RT	Age modifies the association between adjuvant RT and survival in triple-negative breast cancer
Zhai et al. (2020)^30^	Retrospective cohort study	1715 (≥70)	Effects of adjuvant systemic therapy or postoperative RT on overall survival and breast cancer–specific survival T1N0 M0 triple-negative breast cancer	Patients aged ≥70 y favor RT over systemic therapy ≥70 y: Poorer overall survival, breast cancer–specific survival (*P < .*001) RT improves overall survival, breast cancer–specific survival in patients aged ≥70 y	Consider RT for postoperative triple-negative breast cancer in patients aged ≥70 y with T1N0M0 triple-negative breast cancer based on the evaluation of health status

Abbreviation: RT = radiation therapy.

Where reported, RT in the included studies primarily referred to whole-breast irradiation following breast-conserving surgery. Some studies, however, also included postmastectomy RT, especially in node-positive disease. Not all studies clearly specified target volumes such as chest wall, regional node levels with or without internal mammary, whether a boost was applied (eg, a tumor bed boost or a scar boost), or dose and fractionation. All these are known to be significant for outcomes (breast cancer outcomes and toxicity).^34^^,^^35^

Six of these studies reported improvements in overall survival and breast cancer–specific survival.^4^^,^^29-32^^,^^36^^,^^37^ For instance, Tang et al.^4^ saw age and RT as independent prognostic factors for overall survival while demonstrating RT’s role in improving breast cancer–specific survival. Xu et al.^29^ developed a nomogram to stratify older women with triple-negative breast cancer by RT risk, suggesting overall survival and breast cancer–specific survival benefits in high-risk patients, overall survival benefits only in intermediate-risk groups, and no observed benefits in low-risk patients (risk groups defined based on a numerical categorization of age, race, ERBB2 status, T stage, and N stage). These findings underscore the importance of tailoring RT recommendations to individual risk profiles.

Age-related disparities in treatment were also noted, with RT more frequently withheld in older women with triple-negative breast cancer who have more comorbidities or following systemic therapy.^31^ Even after propensity score matching, RT omission was an independent factor correlated with poorer overall survival. In addition, breast-conserving surgery without RT saw a doubling in future mastectomy rates due to locoregional recurrence. Older patients were more likely to accept postoperative RT than systemic therapy, however, a factor that could partially rationalize the observed changes in overall survival.^30^

One study that had a smaller sample size (*n* = 1138) observed no survival benefit from RT in older women with triple-negative breast cancer.^36^ Differences in findings could be attributed to limited statistical power, variations in tumor subtypes, or regional differences in treatment regimens.

### Systemic therapy

Eleven retrospective cohort studies explored the associations between systemic therapy and outcomes in older women with triple-negative breast cancer (Table 3; Table SB3).^32^^,^^38-40^ Collectively, these studies included more than 57 000 patients aged 65 years and older. Potential data duplication is possible due to 6 studies using the SEER database^4^^,^^30^^,^^40-43^ and potential overlap with the National Cancer Database.^32^^,^^39^^,^^44^ Most research focused on adjuvant systemic therapy, with only 1 study investigating neoadjuvant therapy.^39^

**Table 3.** Papers primarily focused on systemic therapy interventions in older women with triple-negative breast cancer.

Author (year)	Study type	Patients meeting our study criteria, No. (age, y)	Treatment regimen investigated	Findings	Investigators’ recommended outcomes
Tang et al (2022)^4^	Retrospective cohort study plus nomogram	4761 (≥70)	Impact of RT or systemic therapy after surgery on long-term overall survival and breast cancer–specific survival	Systemic therapy improves 3-, 5-, 7-y overall survival (*P < .*0001), limited breast cancer–specific survival benefit (*P = .*17) No systemic therapy or RT worsens overall survival, breast cancer–specific survival (*p* < .0001) Age, systemic therapy, and RT predict overall survival (*P < .*001)	Results support active triple-negative breast cancer management in older patients
Janeva et al. (2020)^38^	Retrospective cohort study plus propensity score matching	1130 (≥70)	Adjuvant systemic therapy	Adjuvant systemic therapy improves 5-y breast cancer–specific survival, overall survival (*P < .*0001), persisting after propensity score matching (*P = .*029) Cox regression: 5-y breast cancer–specific survival, overall survival (*P = .*013, *P = .*0035)	Older women with triple-negative breast cancer benefit from adjuvant systemic therapy
Valachis et al. (2021)^46^	Retrospective cohort study	413 (≥70)	Systemic therapy treatment patterns, risk factors for hospitalization, and causes of death in older women with triple-negative breast cancer	Age and comorbidity associated with lower systemic therapy use Systemic therapy use linked to increased hospitalization risk Higher stage, comorbidity increase breast cancer mortality Age, advanced stage, and comorbidity independently raise overall mortality	Need to tailor triple-negative breast cancer treatment for older patients No survival benefit observed with systemic therapy
Guo et al. (2023)^40^	Retrospective cohort study plus inverse probability treatment weighting	4610 (≥70)	Effects of adjuvant systemic therapy	Systemic therapy yields better 5-y breast cancer–specific survival, overall survival (*p* < .001) T1ab patients gain no systemic therapy benefit (*P* > .050) In >80 y age group, overall survival improves	Older women with triple-negative breast cancer benefit from adjuvant systemic therapy in survival Caution needed in de-escalated treatment for T1ab patients Particularly consider physical status for systemic therapy in patients aged >80 y
M Xiu et al. (2022)^29^	Retrospective cohort study	177 (≥65)	Current systemic therapy options in older women with early-stage triple-negative breast cancer	Systemic therapy (≥1 cycle) boosts breast cancer–specific survival, overall survival (*P = .*046, *P = .*029) Regimen options differ by age, stage Anthracycline and taxane for patients aged 65-69 y, stage II-III disease ≥70 y: carboplatin based, often TCb	Older women with triple-negative breast cancer showed improved breast cancer–specific survival and overall survival with systemic therapy, suggesting a need for individualized regimes TCb regimen may be feasible when patients cannot tolerate anthracycline
Brown et al. (2022)^39^	Retrospective cohort study plus propensity score matching	12 090 (≥70 )	Effects of primary systemic therapy in patients aged ≥70 y and ≥80 y with ERBB2-positive or triple-negative breast cancer	Primary systemic therapy benefits overall survival in triple-negative breast cancer Surgery omission after primary systemic therapy, poor overall survival Careful selection for primary systemic therapy crucial Primary systemic therapy improves overall survival in both groups (*P < .*001)	Primary systemic therapy plus surgery is effective for both groups Careful selection is crucial for optimizing outcomes in this population
Crozier et al. (2020)^32^	Retrospective cohort study plus propensity score matching	16 062 (≥70)	Addition of systemic therapy to local therapy on overall survival in older women with triple-negative breast cancer	Systemic therapy improves overall survival (*P < .*0001), persisting after adjustment for node status and comorbidity No clear age limit for systemic therapy benefit	Data support considering systemic therapy and RT in treating women aged ≥70 y with triple-negative breast cancer
Schreiber et al. (2021)^43^	Retrospective cohort study	3348 (≥66)	Effects of anthracycline and taxane vs taxane-based systemic therapy regimen on cancer-specific survival and overall survival in node-negative older women with triple-negative breast cancer	Patients aged ≥76 y less likely to receive systemic therapy (*P < .*0001) Older women, less likely candidates for anthracycline and taxane (*P < .*0001) Adjuvant systemic therapy improves cancer-specific survival, overall survival Taxane better than anthracycline and taxane for cancer-specific survival, overall survival	Consider use of systemic therapy in older patients who are expected to obtain benefit from taxane, with a pause in the use of anthracycline and taxane in this population
Roy et al. (2023)^42^	Retrospective cohort study	1106 (≥66)	Evaluate the benefit of adjuvant anthracycline and taxane vs taxane in node-positive older women with triple-negative breast cancer	Systemic therapy improves 3-y breast cancer–specific survival, overall survival (*P < .*01) Anthracycline and taxane and taxane have similar outcomes (*P = .*80, *P = .*79) No significant heart failure difference (*P = .*45) Older patients benefit from anthracycline and taxane (*P = .*02) Trend toward worse cancer-specific survival in 1-3 nodes with anthracycline and taxane (*P = .*05)	Older women with triple-negative breast cancer less likely to undergo adjuvant systemic therapy, despite it showing a clear survival benefit Anthracycline and taxane lacks survival benefit but could be beneficial with ≥4 lymph nodes
Roy et al. (2024)^41^	Retrospective cohort study	2215 (≥66)	Incidence of major adverse cardiovascular events in triple-negative breast cancer after adjuvant systemic therapy	Patients aged 66-75 y on anthracycline and taxane (*P < .*01) No higher risk in anthracycline and taxane for: Myocardial infarction (*P = .*91) Heart failure (*P < .*01) Potentially fatal arrhythmia (*P = .*12) Cerebrovascular accident (*P = .*20) No difference in overall survival, cancer-specific survival for cardiac outcomes (taxane vs anthracycline and taxane)	Age may serve as a predictor for worse survival for systemic therapy once a cardiac event occurs
Zhai et al. (2020)^30^	Retrospective cohort study	1715 (≥70)	Effects of adjuvant systemic therapy or postoperative RT on overall survival and breast cancer–specific survival in T1N0 M0 triple-negative breast cancer	Older patients favor RT over systemic therapy ≥70 y: poorer overall survival, breast cancer–specific survival (*P < .*001) Adjuvant systemic therapy improves breast cancer–specific survival and overall survival RT improves overall survival, breast cancer–specific survival in patients aged ≥70 y	Consider RT for postoperative patients aged ≥70 y with triple-negative breast cancer patients and T1N0M0 triple-negative breast cancer based on the evaluation of health status

Abbreviation: RT = radiation therapy.

All included studies demonstrated associations between adjuvant systemic therapy and improved outcomes. Four studies identified better overall survival,^4^^,^^30^ while 2 also reported improvements in breast cancer–specific survival,^32^^,^^38^ observations that persisted even after propensity score matching. Tang et al.^4^ saw systemic therapy receipt as an independent favorable prognostic factor for overall survival.

Systemic therapy regimens varied by age, with younger patients (<70 years) more likely to receive anthracycline and taxane–based regimens and older patients (≥70 years) more often receiving carboplatin-based regimens.^45^ No substantial differences were observed in recurrence-free survival or breast cancer–specific survival between regimens within age groups.^45^ Schreiber et al.^43^ reported that patients 76 years of age and older were less likely to receive anthracycline and taxane–based regimens, likely due to concerns about treatment toxicity.

Reduced systemic therapy use was independently associated with increased age and comorbidities, possibly reflecting higher hospitalization risks during and after treatment.^30^^,^^40^^,^^43^^,^^46^ Guo et al.^40^ found improvements in overall survival and breast cancer–specific survival with adjuvant systemic therapy in older women with triple-negative breast cancer, although breast cancer–specific survival benefits were not observed in early-stage tumors (T1a/b) or in patients older than 80 years of age. These findings suggest caution when administering systemic therapy, emphasizing patient fitness and frailty over age.^43^ Updated guidelines supporting individualized treatment approaches have increased systemic therapy uptake since 2012.^21^ Recent studies also highlight the absolute benefits of systemic therapy for small triple-negative breast cancer tumors, showing improvements in 5-year breast cancer–specific survival.^47^

Interestingly, completing 1 or more cycles of adjuvant systemic therapy was associated with improved overall survival and breast cancer–specific survival across all age groups. This implies that less intense, carboplatin-based regimens may benefit patients, even if treatment is not completed, as highlighted in the cohort studied by Guo et al.^40^^,^^48^

One study investigating primary systemic therapy in older patients reported improved overall survival among individuals who received primary systemic therapy compared with patients who did not.^39^ Further research is needed, however, to determine whether primary systemic therapy can benefit older women with triple-negative breast cancer currently deemed unfit for treatment, underscoring the need for individualized approaches to therapy selection.

### Differences in treatment/outcomes due to the intrinsic tumor biology of triple-negative breast cancer

The final group of 11 papers (Table 4; Table SB4) focused on treatment disparities, tumor characteristics, and outcomes in older women with triple-negative breast cancer. All were retrospective cohort studies,^10^^,^^49-58^ with potential overlap among 3 studies using the SEER database (2010-2014).^21^^,^^23^^,^^49^

**Table 4.** Papers primarily focused on disparities in outcomes of different interventions in older women with triple-negative breast cancer.

Author (year)	Study type	Patients meeting our study criteria, No. (age, y)	Treatment regimen investigated	Findings	Investigators’ recommended outcomes
Drapalik et al. (2022)^10^	Retrospective cohort study	36 042 (≥70)	Disparities in treatment and outcomes based on age	Adjuvant systemic therapy, neoadjuvant systemic therapy improve survival (*P < .*0001) Poorer survival with unilateral mastectomy, no surgery (*P < .*0001) RT improves survival in all ages (*P < .*05) In oldest patients, omission of systemic therapy, axillary surgery, RT	Multimodal therapy options should be considered for all patients, irrespective of age
Kaplan et al. (2017)^49^	Retrospective cohort study	159 (≥65)	Surgery, systemic therapy, and RT treatment outcomes in older women with triple-negative breast cancer	≥75 y: surgery, RT common (48%) ≥75 y: more likely to have mastectomy RT similar across age groups ≥75 y: less likely to be on doxorubicin regimen (*P < .*001) Systemic therapy completion declines with age (*P = .*001) Age not significant predictor in Cox model	In patients aged ≥75 y with triple-negative breast cancer, lower treatment levels yield outcomes similar to younger patients
Bulut et al. (2015)^50^	Retrospective cohort study	58 (≥60)	Histopathologic features and survival of older vs younger patients with triple-negative breast cancer	More comorbid diseases in older patients (*P = .*001) Adjuvant RT differs between age groups (*P = .*001) No diminishing systemic therapy benefit with age No difference in DFS, overall survival (*P = .*914, *P = .*939)	Similar survival in older and younger patients with triple-negative breast cancer suggests common tumor characteristics
Zhu et al. (2015)^51^	Retrospective cohort study	2207 (≥70)	Early breast cancer–specific survival and overall survival patterns (within 2 y of diagnosis) in older and younger patients with triple-negative breast cancer	>70 y: Predicts poor breast cancer–specific survival, overall survival (*P < .*001) Older women with triple-negative breast cancer: benign biological features (*P < .*001), less lymph node metastasis (*P < .*001), earlier TNM stage (*P < .*001), better differentiation (*P < .*001) Older women with triple-negative breast cancer undergoing surgery had better immediate breast cancer–specific survival (*P < .*001) Underuse of curative treatment, especially RT, more common in older women with stage II or III disease Surgery alone: age-related survival gap (*P < .*001)	Denying standard-of-care anticancer treatment to this cohort merely based on age is not fully justified
Spoer et al. (2023)^52^	Retrospective cohort study	21 (≥80)	Differences in outcomes based on level of care in older women with triple-negative breast cancer	No significant mortality differences between standard of care and insufficient care groups Treatment deferral linked to poorer functional status; ECOG-ACRIN III significantly associated with deferral (*P = .*0140)	This pilot study emphasizes that standard-of-care treatment offerings should be directed by tumor biology and individual patient profiles rather than age alone
Qiu et al. (2016)^53^	Retrospective cohort study	66 (≥60)	Investigate the clinicopathological features, recurrence, metastasis, treatment methods, and prognosis of the younger vs older women with triple-negative breast cancer	Older group had higher survival Older group received less RT, systemic therapy Significant differences in RT, systemic therapy (*P < .*001)	The older and younger patients with triple-negative breast cancer may belong to different biological subtypes More conservative and cautious attitude in choosing the postoperative adjuvant treatment for older women with triple-negative breast cancer
Kozak et al. (2019)^54^	Retrospective cohort study	4221 ( ≥ 70)	Postoperative RT or adjuvant systemic therapy in women with triple-negative breast cancer	Women aged ≥70 y received less systemic therapy, RT No cancer-specific mortality difference in adjuvant treatment Cancer-specific mortality difference diminished with both systemic therapy and RT Older patients equally chose conservation, mastectomy	Older women with triple-negative breast cancer benefit from more aggressive therapy, and that undertreatment rather than age itself is in part responsible for worse outcomes in this patient population
Tzikas et al. (2020)^55^	Retrospective cohort study	96 (>74)	Biology, recurrence rate, metastatic patterns, and survival times in triple-negative breast cancer between older and young patients	Adjusting for systemic therapy, survival differences vanished Older patients favored mastectomy, less axillary surgery Younger patients received more systemic therapy (*P = .*0005) Systemic therapy type varied with age (*P = .*004) Older patients received less RT	Older women with triple-negative breast cancer in good general condition could benefit from adjuvant systemic therapy, and less toxic regimens can be an efficient option
Tan et al. (2023)^56^	Retrospective cohort study	2610 (≥75)	Age as a prognostic factor in triple-negative breast cancer	Overall survival and breast cancer–specific survival significantly lower in older patients Older patients with triple-negative breast cancer had lower rates of surgery Excluding treatments increased hazard ratio Poor prognosis partly due to treatment disparities Age is an independent risk factor for prognosis in patients with triple-negative breast cancer	Older women with triple-negative breast cancer receive less active treatment than their younger counterparts
Syed et al. (2014)^57^	Retrospective cohort study	127 (≥70)	Analyze the biology and clinical outcome of older vs younger patients with triple-negative breast cancer	Fewer older patients received systemic therapy Triple-negative breast cancer in older women is less aggressive Similar outcomes irrespective of age	Biological differences in the tumors could explain why older women did not have worse outcomes despite less treatment
Honma et al. (2021)^58^	Retrospective cohort study	75 (≥75)	Clinicopathological characteristics of older vs younger patients with triple-negative breast cancer	Older women with triple-negative breast cancer received less adjuvant therapy Older group: higher androgen receptor, lower CK5/6, tumor-infiltrating lymphocytes Androgen receptor positivity benefits older patients Tumor-infiltrating lymphocyte status not influential in outcomes Patients with androgen receptor–positive triple-negative breast cancer may avoid systemic therapy No age-based differences in histology	Androgen receptor positivity was a predictor of decreased recurrence in older women with triple-negative breast cancer Androgen receptor–positive tumors but not tumors with high levels of tumor-infiltrating lymphocytes were associated with a favorable clinical outcome in older patients, suggesting the importance of examining androgen receptors

Abbreviations: DFS = disease-free survival; RT = radiation therapy.

Although defined by the absence of estrogen receptor, progesterone receptor, and ERBB2 expression, genomic analyses have revealed intrinsic triple-negative breast cancer subtypes, including basal-like (BL1 and BL2), mesenchymal, luminal androgen receptor, and immunomodulatory subtypes.^16^^,^^17^ Among these, basal-like triple-negative breast cancer constitutes the majority (approximately 80%) and is associated with higher proliferative rates and poorer prognoses.^6^ In contrast, luminal androgen receptor–positive triple-negative breast cancer demonstrates a less aggressive phenotype with lower proliferation indices and better outcomes. Interestingly, studies suggest that older women with triple-negative breast cancer exhibit a higher frequency of luminal androgen receptor and mesenchymal-like subtypes, which could partly explain the observed differences in tumor aggressiveness compared with younger patients.^57^ The role of androgen receptors in triple-negative breast cancer is particularly relevant because androgen receptor–positive triple-negative breast cancer is associated with reduced sensitivity to conventional systemic therapies but may respond to antiandrogenic treatments.^51^ These molecular differences may contribute to variations in treatment response and survival outcomes between older and younger women with triple-negative breast cancer and warrant further investigation into age-specific therapeutic strategies.

Triple-negative breast cancer in older women often shows less aggressive biological traits, such as lower Ki67 levels, more wild-type p53 expression, and higher Bcl2 expression. Despite these differences, clinical outcomes between women 70 years of age and older and women younger than 70 years of age showed no statistically significant variation at a median follow-up of 46 months.^57^ Honma et al.^58^ reported higher androgen receptor positivity, lower CK5/6, and reduced tumor-infiltrating lymphocytes in older patients. Androgen receptor–positive tumors were associated with better DFS but poorer responsiveness to systemic therapy. This finding indicates that these patients may benefit more from tailored therapies, such as tumor-infiltrating lymphocyte–based treatments or programmed cell death 1 ligand 1 inhibitors.^59^

Age-related disparities in treatment were evident across studies. Patients aged 75 years or older were less likely to receive adjuvant systemic therapy and more likely to undergo mastectomy than women younger than 75 years of age.^49^ Age was not, however, found to be a substantial predictor of breast cancer mortality in Cox regression models. Similarly, Kozak et al.^54^ and Bulut et al.^50^ suggested that tumor differences, rather than age, should guide treatment decisions.

Spoer et al.^52^ observed that most women receiving standard care underwent breast-conserving surgery plus sentinel node biopsy, while mastectomy was more prevalent among women with insufficient or deferred care. Despite similar clinical indications, adjuvant therapies were substantially underutilized in the deferred care group. Importantly, no statistically significant mortality differences were found between groups, suggesting that chronological age should not be the sole determinant of treatment.

Undertreatment of older women with triple-negative breast cancer was highlighted as a key issue. Qiu et al.^53^ reported improved 5-year DFS and overall survival in older women, even with lower adjuvant therapy rates than younger patients. One study, however, initially found shorter survival, recurrence-free survival, and breast cancer–specific survival in older women, although these differences became statistically insignificant after adjusting for adjuvant or neoadjuvant systemic therapy.^55^ Tan et al.^56^ and Drapalik et al.^60^ further corroborated these findings, highlighting the importance of personalized treatment approaches based on tumor biology and patient fitness rather than chronological age.

## Discussion

This review examined real-world treatment practices and outcomes in older women with early-stage triple-negative breast cancer, focusing on disparities in treatment receipt, survival outcomes by age and therapy type, and evidence supporting multimodal care. Triple-negative breast cancer is aggressive and prone to early recurrences,^61^ particularly within 2 to 3 years. Effective management is associated with the potential to prevent recurrence within the patient’s lifespan, which could substantially improve QOL. This systematic review examined treatment approaches and outcomes in the context of the rising incidence of triple-negative breast cancer in older women because of an aging population. Our study is not a patient-level meta-analysis, but the real-world data provides valuable insights.

Although most included studies are retrospective and subject to inherent biases, the evidence suggests an association between breast-conserving surgery plus RT and improved survival in suitable patients. Notably, breast-conserving surgery can often be performed under local anesthesia, which may benefit patients at higher risk of complications associated with general anesthesia.^62^ Older patients are frequently deemed unfit for surgery when alternatives such as hormone therapies (eg, tamoxifen) are available for estrogen receptor–positive cancers. For estrogen receptor–negative cancers, where alternate treatments are more limited, these same patients are often assessed as fit for surgery, raising the possibility of unconscious bias in clinical decision making.^63^

A review of oncoplastic breast-conserving surgery uptake in women older than 65 years of age revealed a low adoption rate of 10.8% (*n* = 61) across 10 studies (*n* = 567), with limited clarification of the underlying reasons^64^; 1 case series in this review mentioned T3 tumors (extreme oncoplastic surgery). A recent meta-analysis by the senior author’s group found oncoplastic breast-conserving surgery useful following partial or poor response to primary systemic therapy.^65^ In older women with triple-negative breast cancer, the uptake is likely to be similar or potentially less due to downsizing in individuals who can safely receive primary systemic therapy.

Another review revealed that only 10% of women older than 65 years of age underwent immediate postmastectomy breast reconstruction compared with 45% of younger women over 10 studies (2012-2020).^66^ It is worth noting that older women often prefer less invasive interventions, aligning with surgical and anesthesia recommendations aimed at minimizing operative and recovery times.

Regarding adjuvant systemic therapy, the reviewed studies consistently reported improved survival outcomes in treated older women with triple-negative breast cancer. Most chemotherapy data are from adjuvant settings, and neoadjuvant treatment (the current standard) is underrepresented in older women with triple-negative breast cancer. Standard regimens include anthracycline-based, alkylator-based, and taxane-based therapies, with nonanthracycline regimens typically reserved for patients with cardiac comorbidities or lower-risk triple-negative breast cancer variants.^67^ Although breast cancer–specific survival benefits were not observed in specific subgroups, such as patients with T1ab tumors or patients aged 80 years or older, overall survival improvements were evident.^40^ These findings, however, may reflect selection biases in treatment allocation. Systemic therapy use has been identified as an independent prognostic factor in older women with triple-negative breast cancer, yet increasing age and the presence of comorbidities are consistently associated with reduced systemic therapy use.^29^^,^^41-43^ Notably, using at least 1 cycle of any systemic therapy regimen has been linked to improved breast cancer–specific survival,^29^ showing the potential benefits of even reduced-intensity regimens for patients who may not tolerate complete courses of therapy.^42^^,^^43^ Given the increasing prevalence of cardiac comorbidities with age, greater consideration of nonanthracycline regimens in older women with triple-negative breast cancer may be warranted. Primary systemic therapy provided an overall survival benefit to eligible older women with triple-negative breast cancer, reinforcing its potential value in this group.

Data on immunotherapy in older populations are limited. A small case series reported that 3 of 5 patients treated with immune checkpoint inhibitors achieved near-complete pathological responses at surgery, although some experienced therapy-related toxicities.^29^ Currently, PARP inhibitors are approved for use in *BRCA1/2*-mutated breast cancers, but their role beyond this indication is under active investigation. Although limited literature addresses explicitly the use of PARP inhibitors in older women with triple-negative breast cancer, their reduced side effect profile compared with systemic therapy positions them as a promising area for future research.

Age and receipt of RT have been identified as independent prognostic factors for overall survival. However, RT is frequently omitted in older women with triple-negative breast cancer, and this omission is associated with poorer overall survival outcomes.^31^ One study reported that RT provided an overall survival benefit only in high-risk and medium-risk older patients, without demonstrating a statistically significant benefit in breast cancer–specific survival for lower-risk patients.^29^ This finding suggests that the omission of RT might be justified in some cases, potentially reflecting underlying factors such as tumor biology or cohort selection. Nevertheless, the existing literature generally supports the benefit of adjuvant RT in older women with triple-negative breast cancer.^29^ Most included studies reported on whole-breast radiation after breast-conserving surgery; however, specific details about target volumes (eg, locoregional vs chest wall or nodal RT) were inconsistently reported, limiting subgroup interpretation.

New fractionation schemes, such as the 5-fraction regimen, may offer substantial advantages for older women with triple-negative breast cancer who require breast or chest wall irradiation because they reduce the duration of treatment without compromising efficacy.^68^^,^^69^ This regimen is currently being evaluated for regional nodal irradiation.^70^ Highly proliferative, grade 3 tumors often necessitate an additional tumor bed boost. In the Fast Forward trial, the triple-negative breast cancer cohort was limited, and although a tumor bed boost was permitted, its application was left to the treating physician’s discretion.^68^^,^^69^ The 20-year update of the European Organisation for Research and Treatment of Cancer Boost no-boost trial demonstrated the most significant absolute benefit of a tumor bed boost in high-grade tumors and patients younger than 50 years of age.^71^

Whole-breast RT is recommended for all triple-negative breast cancer subtypes due to their high local recurrence rates. The 15-fraction regimen, delivering 40 Gy in 15 fractions and a tumor bed boost, should be considered for grade 3, basal-like, triple-negative breast cancer cases, particularly in patients with close or positive surgical margins or in patients without systemic therapy because it improves local control. The 5-fraction 26-Gy regimen is effective and convenient for frail women with small, early-stage tumors and is increasingly considered for select mastectomy patients. It reduces treatment burden and hospital visits while maintaining local control. This approach is especially valuable for minimizing strain and exposure in vulnerable populations, but basal-like triple-negative breast cancer has been shown to exhibit greater radioresistance.^72^ For older patients with clinically significant comorbidities, local control may hold greater importance than survival benefits. Furthermore, adverse aesthetic outcomes associated with RT are often long term and may be less relevant for patients with limited overall survival.^73^

Studies on older women with triple-negative breast cancer and their tumors show different outcomes. Although this population is undertreated^20^^,^^49-57^^,^^60^ and tumors are smaller and lower grade, with less metastasis,^20^^,^^50^^,^^51^^,^^56^^,^^57^ 6 papers found no difference in overall survival and breast cancer–specific survival between older and younger patients with triple-negative breast cancer.^20^^,^^50^^,^^52^^,^^54^^,^^55^^,^^57^ One study reported better DFS and overall survival in older women with triple-negative breast cancer, despite undertreatment (receiving less than the guideline-suggested standard of care for the particular tumor subtype).^53^ Two studies initially showed worse breast cancer–specific survival in older women with triple-negative breast cancer,^55^^,^^56^ but this finding became insignificant in 1 and less significant in the other after adjusting for treatment rates, suggesting that undertreatment harms older women with triple-negative breast cancer.^55^

Survival outcomes in older women with triple-negative breast cancer are multifaceted. Favorable tumor characteristics may suggest improved breast cancer–specific survival and overall survival; undertreatment often leads to worse outcomes. Patient fitness plays a major role in treatment decisions. Although tools such as the G8 Geriatric Screening Tool are specifically designed for older adults, the Charlson Comorbidity Index primarily captures comorbidity burden rather than functional status or frailty. Fitter patients are more likely to receive appropriate therapies, leading to better survival, irrespective of treatment. To address this issue, the UK National Audit of Breast Cancer in Older Patients introduced fitness assessments in 2018 to reduce treatment biases in women older than 70 years of age.^74^ The Audit’s 2022 report, however, highlighted that fewer than 2% of patients underwent each assessment component.^75^ Tools such as the Age Gap Decision Tool have also been developed to support informed treatment decisions in women older than 70 years of age, potentially improving outcomes by promoting individualized care.^76^

Yoon et al.^20^ conducted a systematic review of older women with triple-negative breast cancer, examining breast cancer–specific survival concerning surgery, systemic therapy, and RT. The review highlighted variations in tumor characteristics in older patients, such as smaller tumors, lower grades, and fewer lymph node metastases. In addition, decreased utilization of adjuvant therapies in women older than 70 years of age was associated with more than a 2-fold increase in cancer-specific mortality, a finding that aligns with those of this study. Only 2 studies within the review, however, demonstrated improved breast cancer–specific survival with adjuvant therapy, while others reported no statistically significant differences. Yoon et al. proposed potential biological differences in triple-negative breast cancer between older and younger women, a hypothesis further supported by Zhu et al.^51^

Survival outcomes vary, even when accounting for frailty and fitness through regression and propensity matching. This variability may stem from intrinsic differences in tumor molecular characteristics across populations. Tumors in older women with triple-negative breast cancer demonstrate less aggressive markers,^57^ such as androgen receptor positivity, which has been linked to lower recurrence rates and systemic therapy insensitivity.^58^ These findings suggest that such markers could enable future personalized treatment strategies for triple-negative breast cancer.

The limitations of the reviewed studies primarily arise from their retrospective design, the varying age cutoffs used to define “older” populations, and inherent selection biases stemming from registry restrictions. Many databases lack detailed information about systemic therapy regimens and other prognostic factors, such as underlying conditions, family history, surgical margin status, and patient comorbidities. Much of the systemic therapy data are derived from adjuvant data instead of the neoadjuvant setting (current standard of care in triple-negative breast cancer). Although older chemotherapy regimens dominate existing studies, newer treatment strategies (eg, immunotherapy and PARP inhibitors) are being integrated. Moreover, objective indicators of QOL and patient-reported outcome measures are often underreported.

Registry-based studies also face challenges such as underreported therapy and miscoded patient data. Although follow-up durations varied, even shorter follow-up periods can provide valuable insights due to triple-negative breast cancer’s peak recurrence occurring at 2 to 3 years—a timeline shorter than that of other breast cancer subtypes.^7^ This finding highlights the need for timely and effective investigation and treatment of older women with triple-negative breast cancer because recurrences are likely to fall within the average life expectancy of this population.^1^

ERBB2 status has only recently been included in major registries (eg, since 2010 in SEER), complicating the accurate identification of triple-negative breast cancer cases in earlier datasets. As a result, many of the studies included in this review may overlap in study periods and datasets, potentially influencing the independence of their conclusions. Furthermore, identifying studies explicitly stratified by both age and triple-negative breast cancer status was challenging, leading to potential omissions. The inclusion of papers defining “older” as younger than 70 years rather than adhering to the SIOG definition of older than 70 years represents another limitation that may affect the generalizability of the findings. This heterogeneity is the effect of inconsistent definitions pervading the literature. We hope that our paper highlights this fact and encourages the adoption of a consistent age definition for future care and reporting. Future research should conform to the SIOG definition to improve consistency and applicability.

This review highlights consistent undertreatment of older women with triple-negative breast cancer across surgical, RT, and systemic therapy domains. Despite this, survival outcomes often mirrored those of younger patients, particularly when adjusted for tumor biology and comorbidities, suggesting that age alone should not guide treatment de-escalation. Clearer age-specific guidelines and better integration of geriatric assessment tools are needed to personalize care and ensure that fit older adults are not denied effective treatments.

## Supplementary Material

pkaf049_Supplementary_Data

## Data Availability

The data underlying this article are available in the article and in its online supplementary material.

## References

[1] Lemij AA , Bastiaannet E, de Glas NA, et al Breast cancer in the older population: A global challenge—an epidemiological perspective. Ann Breast Surg. 2023;7:17. 10.21037/abs-21-89

[2] Foulkes WD , Smith IE, Reis-Filho JS. Triple-negative breast cancer. N Engl J Med. 2010;363:1938-1948. 10.1056/NEJMra1001389 21067385

[3] Keegan TH , DeRouen MC, Press DJ, Kurian AW, Clarke CA. Occurrence of breast cancer subtypes in adolescent and young adult women. Breast Cancer Res. 2012;14:R55. 10.1186/bcr3156 22452927 PMC3446389

[4] Tang Z , Ji Y, Min Y, et al Prognostic factors and models for elderly (≥70 years old) primary operable triple-negative breast cancer: analysis from the National Cancer Database. Front Endocrinol (Lausanne). 2022;13:856268. 10.3389/fendo.2022.856268 35370936 PMC8969604

[5] You YH , Kim MK, Lee JY. Prognosis and adjusting factors in elderly patients with triple-negative breast cancer: comparing with young and middle age groups. Clin Breast Cancer. 2024;24:e258-e265. 10.1016/j.clbc.2024.01.016 38413338

[6] Carey LA , Perou CM, Livasy CA, et al Race, breast cancer subtypes, and survival in the Carolina Breast Cancer Study. JAMA. 2006;295:2492-2502. 10.1001/jama.295.21.2492 16757721

[7] Dent R , Trudeau M, Pritchard KI, et al Triple-negative breast cancer: clinical features and patterns of recurrence. Clin Cancer Res. 2007;13:4429-4434. 10.1158/1078-0432.CCR-06-3045 17671126

[8] Martin AM , Cagney DN, Catalano PJ, et al Brain metastases in newly diagnosed breast cancer. JAMA Oncol. 2017;3:1069-1077. 10.1001/jamaoncol.2017.0001 28301662 PMC5824221

[9] Li H , Chen Y, Wang X, Tang L, Guan X. T1-2N0M0 triple-negative breast cancer treated with breast-conserving therapy has better survival compared to mastectomy: a SEER population-based retrospective analysis. Clin Breast Cancer. 2019;19:e669-e682. 10.1016/j.clbc.2019.05.011 31375327

[10] Drapalik LM , Estes A, Sarode AL, et al Age disparities in triple-negative breast cancer treatment and outcomes: an NCDB analysis. Surgery. 2022;172:821-830. 10.1016/j.surg.2022.05.026 35927082

[11] Treatment of Triple-negative Breast Cancer n.d. Accessed August 26, 2024. https://www.cancer.org/cancer/types/breast-cancer/treatment/treatment-of-triple-negative.html

[12] Schmid P , Cortes J, Pusztai L, et al; KEYNOTE-522 Investigators. Pembrolizumab for early triple-negative breast cancer. N Engl J Med. 2020;382:810-821. 10.1056/NEJMoa1910549 32101663

[13] Burstein HJ , Curigliano G, Thürlimann B, et al; Panelists of the St Gallen Consensus Conference. Customizing local and systemic therapies for women with early breast cancer: The St Gallen International Consensus Guidelines for treatment of early breast cancer 2021. Ann Oncol. 2021;32:1216-1235. 10.1016/j.annonc.2021.06.023 34242744 PMC9906308

[14] Shepherd JH , Ballman K, Polley M-YC, et al CALGB 40603 (Alliance): long-term outcomes and genomic correlates of response and survival after neoadjuvant chemotherapy with or without carboplatin and bevacizumab in triple-negative breast cancer. J Clin Oncol. 2022;40:1323-1334. 10.1200/JCO.21.01506 35044810 PMC9015203

[15] von Minckwitz G , Schneeweiss A, Loibl S, et al Neoadjuvant carboplatin in patients with triple-negative and HER2-positive early breast cancer (GeparSixto; GBG 66): a randomised phase 2 trial. Lancet Oncol. 2014;15:747-756. 10.1016/S1470-2045(14)70160-3 24794243

[16] Schmid P , Cortés J, Dent RA, et al LBA18 pembrolizumab or placebo plus chemotherapy followed by pembrolizumab or placebo for early-stage TNBC: updated EFS results from the phase III KEYNOTE-522 study. Ann Oncol. 2023;34:S1257. 10.1016/j.annonc.2023.10.008

[17] Lehmann BD , Pietenpol JA, Tan AR. Triple-negative breast cancer: molecular subtypes and new targets for therapy. Am Soc Clin Oncol Educ Book. 2015;e31-e39. 10.14694/EdBook_AM.2015.35.e31 25993190

[18] Dacoregio MI , Vilbert M, Stecca C, Michelon I, Castro C, Amir E. Neoadjuvant PARP inhibitors in patients with early HER2-negative breast cancer harboring BRCA 1/2 germline mutations: a systematic review and meta-analysis. J Clin Oncol. 2023;41:e12611. 10.1200/JCO.2023.41.16_suppl.e12611

[19] Ss S TJ , Hb M EJ. Management of triple-negative breast cancer in older patients: how is it different? Oncology (Williston Park). 2018;32:58-63. 29492945

[20] Yoon J , Knapp G, Quan ML, Bouchard-Fortier A. Cancer-specific outcomes in the elderly with triple-negative breast cancer: a systematic review. Curr Oncol. 2021;28:2337-2345. 10.3390/curroncol28040215 34202498 PMC8293164

[21] National Audit of Breast Cancer in Older Patients (NABCOP). National Audit of Breast Cancer in Older Patients (NABCOP) Annual Report 2020. 2020. https://www.nabcop.org.uk/reports/nabcop-2020-annual-report/

[22] Pembrolizumab for neoadjuvant and adjuvant treatment of triple-negative early or locally advanced breast cancer n.d. https://www.nice.org.uk/guidance/ta851/chapter/1-Recommendations (accessed August 26, 2024). 40587639

[23] Hutchins LF , Unger JM, Crowley JJ, Coltman CA, Albain KS. Underrepresentation of patients 65 years of age or older in cancer-treatment trials. N Engl J Med. 1999;341:2061-2067. 10.1056/NEJM199912303412706 10615079

[24] Page MJ , McKenzie JE, Bossuyt PM, et al The PRISMA 2020 statement: an updated guideline for reporting systematic reviews. BMJ. 2021;372:n71. 10.1136/bmj.n71 33782057 PMC8005924

[25] Sterne JA , Hernán MA, Reeves BC, et al ROBINS-I: a tool for assessing risk of bias in non-randomised studies of interventions. BMJ. 2016;355:i4919. 10.1136/bmj.i4919 27733354 PMC5062054

[26] Mburu W , Kulasingam S, Hodges JS, Virnig BA. Breast-conserving surgery versus mastectomy for older women with triple-negative breast cancer: population-based study. J Comp Eff Res. 2022;11:953-967. 10.2217/cer-2021-0273 35894095

[27] Saifi O , Chahrour MA, Li Z, et al Is breast conservation superior to mastectomy in early stage triple negative breast cancer? Breast. 2022;62:144-151. 10.1016/j.breast.2022.02.006 35182994 PMC8859006

[28] Zhong Y , Xu Y, Zhou Y, et al Breast-conserving surgery without axillary lymph node surgery or radiotherapy is safe for HER2-positive and triple negative breast cancer patients over 70 years of age. Breast Cancer Res Treat. 2020;182:117-126. 10.1007/s10549-020-05686-3 32430680

[29] Xu L , Zhou C, Qiu J, Lv Q, Du Z. Can radiotherapy after breast-conserving surgery be omitted in elderly patients with early-stage, hormone-receptor negative breast cancer? A population-based study and proposed nomogram. Adv Ther. 2022;39:4707-4722. 10.1007/s12325-022-02279-y 35953665

[30] Zhai Z , Zheng Y, Yao J, et al Evaluation of adjuvant treatments for T1 N0 M0 triple-negative breast cancer. JAMA Netw Open. 2020;3:e2021881. 10.1001/jamanetworkopen.2020.21881 33211105 PMC7677762

[31] Haque W , Verma V, Hsiao K-Y, et al Omission of radiation therapy following breast conservation in older (≥70 years) women with T1-2N0 triple-negative breast cancer. Breast J. 2019;25:1126-1133. 10.1111/tbj.13443 31273872

[32] Pezzi CM , Crozier JA, Pezzi TA, et al Addition of chemotherapy to local therapy in women aged 70 years or older with triple-negative breast cancer: a propensity-matched analysis. Lancet Oncol. 2020;21:1611-1619. 33271091 10.1016/S1470-2045(20)30538-6

[33] Bhoo-Pathy N , Verkooijen HM, Wong F-Y, et al Prognostic role of adjuvant radiotherapy in triple-negative breast cancer: a historical cohort study. Int J Cancer. 2015;137:2504-2512. 10.1002/ijc.29617 26018878

[34] Kaidar-Person O , Fortpied C, Hol S, et al EORTC Radiation Oncology and Breast Cancer Groups. The association of internal mammary and medial supraclavicular lymph node radiation technique with clinical outcomes: results from the EORTC 22922/10925 randomised trial. Radiother Oncol. 2022;172:99-110. 10.1016/j.radonc.2022.05.006 35568284

[35] Lee SF , Kennedy SKF, Caini S, et al Randomised controlled trials on radiation dose fractionation in breast cancer: systematic review and meta-analysis with emphasis on side effects and cosmesis. BMJ. 2024;386:e079089. 10.1136/bmj-2023-079089 39260879 PMC11388113

[36] Algan O , Zhao YD, Herman T. Radiotherapy in patients 70 years and older with triple-negative breast cancer. Clin Breast Cancer. 2016;16:e99-e106. 10.1016/j.clbc.2016.05.011 27292180

[37] Haque W , Kee Yuan DM, Verma V, et al Radiation therapy utilization and outcomes for older women with breast cancer: impact of molecular subtype and tumor grade. Breast. 2017;35:34-41. 10.1016/j.breast.2017.06.011 28646722

[38] Janeva S , Zhang C, Kovács A, et al Adjuvant chemotherapy and survival in women aged 70 years and older with triple-negative breast cancer: a Swedish population-based propensity score-matched analysis. Lancet Healthy Longev. 2020;1:e117-e124. 10.1016/S2666-7568(20)30018-0 36094184

[39] Brown L , Naffouje SA, Sam C, Laronga C, Catherine Lee M. Neoadjuvant systemic therapy in geriatric breast cancer patients: a National Cancer Database (NCDB) analysis. Breast Cancer Res Treat. 2022;196:441-451. 10.1007/s10549-022-06751-9 36207620

[40] Guo Q , Lan T, Lu Y, et al Effectiveness of adjuvant chemotherapy for elderly patients with triple-negative breast cancer. Biomol Biomed. 2023;23:502-509. 10.17305/bjbms.2022.8163 36408954 PMC10171436

[41] Roy S , Lakritz S, Schreiber AR, et al Major cardiovascular adverse events in older adults with early-stage triple-negative breast cancer treated with adjuvant taxane + anthracycline versus taxane-based chemotherapy regimens: a SEER-Medicare study. Eur J Cancer. 2024;196:113426. 10.1016/j.ejca.2023.113426 38000217 PMC11451478

[42] Roy S , Lakritz S, Schreiber AR, et al Clinical outcomes of adjuvant taxane plus anthracycline versus taxane-based chemotherapy regimens in older adults with node-positive, triple-negative breast cancer: a SEER–Medicare study. Eur J Cancer. 2023;185:69-82. 10.1016/j.ejca.2023.02.014 36965330 PMC11918260

[43] Schreiber AR , Kagihara J, Eguchi M, et al Evaluating anthracycline + taxane versus taxane-based chemotherapy in older women with node-negative triple-negative breast cancer: a SEER-Medicare study. Breast Cancer Res Treat. 2022;191:389-399. 10.1007/s10549-021-06424-z 34705147 PMC8763743

[44] Yolcu Y , Wahood W, Kerezoudis P, Alvi MA, Habermann EB, Bydon M. Primary central nervous system tumors: comparing two national cancer registries. World Neurosurg. 2019;128:e719-e731. 10.1016/j.wneu.2019.04.247 31077903

[45] Xiu M , Zhang P, Li Q, et al Chemotherapy decision-making and survival outcomes in older women with early triple-negative breast cancer: evidence from real-world practice. Front Oncol. 2022;12:867583. 10.3389/fonc.2022.867583 35574419 PMC9097590

[46] Valachis A , Nyström P, Fredriksson I, Wennstig AK, Ahlgren J. Treatment patterns, risk for hospitalization and mortality in older patients with triple negative breast cancer. J Geriatr Oncol. 2021;12:212-218. 10.1016/j.jgo.2020.09.004 32928712

[47] Tarantino P , Leone J, Vallejo CT, et al Prognosis and trends in chemotherapy use for patients with stage IA triple-negative breast cancer (TNBC): a population-based study. J Clin Oncol. 2023;41:510-510. 10.1200/JCO.2023.41.16_suppl.510

[48] Xiu M , Zhang P, Li Q, et al Chemotherapy decision-making and survival outcomes in older women with early triple-negative breast cancer: evidence from real-world practice. Front Oncol. 2022;12:867583. 10.3389/fonc.2022.867583 35574419 PMC9097590

[49] Kaplan HG , Malmgren JA, Atwood MK. Triple-negative breast cancer in the elderly: prognosis and treatment. Breast J. 2017;23:630-637. 10.1111/tbj.12813 28485826

[50] Bulut N , Altundag K. Excellent clinical outcome of triple-negative breast cancer in younger and older women. J Buon. 2015;20:1276-1281. 26537075

[51] Zhu W , Perez EA, Hong R, Li Q, Xu B. Age-related disparity in immediate prognosis of patients with triple-negative breast cancer: a population-based study from SEER cancer registries. PLoS One. 2015;10:e0128345. 10.1371/journal.pone.0128345. 26020519 PMC4447406

[52] Spoer DL , Ghyasi N, Thorson TL, et al Octogenarians with triple negative breast cancer. Ann Breast Surg. 2024;8:16-16. 10.21037/abs-23-58

[53] Qiu JD , Xue XY, Li R, Wang JD. Clinicopathological features and prognosis of triple-negative breast cancer: a comparison between younger (<60) and elderly (≥60) patients. Eur J Cancer Care. 2016;25:1065-1075. 10.1111/ecc.12346 26122025

[54] Kozak MM , Xiang M, Pollom EL, Horst KC. Adjuvant treatment and survival in older women with triple negative breast cancer: a Surveillance, Epidemiology, and End Results analysis. Breast J. 2019;25:469-473. 10.1111/tbj.13251 30925635

[55] Tzikas AK , Nemes S, Linderholm BK. A comparison between young and old patients with triple-negative breast cancer: biology, survival and metastatic patterns. Breast Cancer Res Treat. 2020;182:643-654. 10.1007/s10549-020-05727-x 32524352 PMC7320950

[56] Tan H , Fu D. Influence of advanced age on the prognosis of triple-negative breast cancer patients: a Surveillance, Epidemiology, and End Results-based study. J Cancer Res Ther. 2023;19:S0. 10.4103/jcrt.jcrt_90_21 37147967

[57] Syed BM , Green AR, Nolan CC, Morgan DAL, Ellis IO, Cheung KL. Biological characteristics and clinical outcome of triple negative primary breast cancer in older women—comparison with their younger counterparts. PLoS One. 2014;9:e100573. 10.1371/journal.pone.0100573 24999743 PMC4085072

[58] Honma N , Ogata H, Yamada A, et al Clinicopathological characteristics and prognostic marker of triple-negative breast cancer in older women. Hum Pathol. 2021;111:10-20. 10.1016/j.humpath.2021.01.005 33548251

[59] Debien V , De Caluwé A, Wang X, et al Immunotherapy in breast cancer: an overview of current strategies and perspectives. NPJ Breast Cancer. 2023;9:7. 10.1038/s41523-023-00508-3 36781869 PMC9925769

[60] Drapalik LM , Estes A, Sarode AL, et al Age disparities in triple-negative breast cancer treatment and outcomes: an NCDB analysis. Surgery (United States). 2022;172:821-830. 10.1016/j.surg.2022.05.026 35927082

[61] Lowery AJ , Kell MR, Glynn RW, Kerin MJ, Sweeney KJ. Locoregional recurrence after breast cancer surgery: a systematic review by receptor phenotype. Breast Cancer Res Treat. 2012;133:831-841. 10.1007/s10549-011-1891-6 22147079

[62] Ghosh A , Sivakanthan T, Forouhi P, Kleidi E, Agrawal A. Surgical outcomes of breast conserving surgery performed under local versus general anaesthesia. Eur J Surg Oncol. 2023;49:e257. 10.1016/j.ejso.2023.03.156

[63] Garreffa E , Agrawal A. Oncoplastic breast surgery in elderly primary breast cancer: time to serve more surgically? Eur J Plast Surg. 2022;46:215-218. 10.1007/s00238-022-02009-1

[64] Chia Z , Lee RXN, Cardoso MJ, Cheung KL, Parks RM. Oncoplastic breast surgery in older women with primary breast cancer: systematic review. Br J Surg. 2023;110:1309-1315. 10.1093/bjs/znad161 37310128 PMC10480033

[65] Ahmed GA , Baron DH, Agrawal A. Oncologic and cosmetic outcomes of oncoplastic breast-conserving surgery after neoadjuvant systemic therapy: systematic review and meta-analysis. Breast Cancer Res Treat. 2025;209:229-252. 10.1007/s10549-024-07566-6 39673644

[66] Lee RXN , Cardoso MJ, Cheung KL, Parks RM. Immediate breast reconstruction uptake in older women with primary breast cancer: systematic review. Br J Surg. 2022;109:1063-1072. 10.1093/bjs/znac251 35909248 PMC10364779

[67] Gennari A , André F, Barrios CH, et al; ESMO Guidelines Committee. Electronic address: Clinicalguidelines@esmo.org. ESMO Clinical Practice Guideline for the diagnosis, staging and treatment of patients with metastatic breast cancer. Ann Oncol. 2021;32:1475-1495. 10.1016/j.annonc.2021.09.019 34678411

[68] Brunt AM , Haviland JS, Sydenham M, et al Ten-year results of FAST: a randomized controlled trial of 5-fraction whole-breast radiotherapy for early breast cancer. J Clin Oncol. 2020;38:3261-3272. 10.1200/JCO.19.02750 32663119 PMC7526720

[69] Meattini I , Becherini C, Boersma L, et al European Society for Radiotherapy and Oncology Advisory Committee in Radiation Oncology Practice consensus recommendations on patient selection and dose and fractionation for external beam radiotherapy in early breast cancer. Lancet Oncol. 2022;23:e21-e31. 10.1016/S1470-2045(21)00539-8 34973228

[70] Wheatley D , Haviland J, Patel J, et al OC-0101 First results of FAST-Forward phase 3 RCT nodal substudy: 3-year normal tissue effects. Radiother Oncol. 2022;170:S75-S76. 10.1016/S0167-8140(22)02477-X

[71] Bartelink H , Maingon P, Poortmans P, et al; European Organisation for Research and Treatment of Cancer Radiation Oncology and Breast Cancer Groups. Whole-breast irradiation with or without a boost for patients treated with breast-conserving surgery for early breast cancer: 20-year follow-up of a randomised phase 3 trial. Lancet Oncol. 2015;16:47-56. 10.1016/S1470-2045(14)71156-8 25500422

[72] Kyndi M , Sørensen FB, Knudsen H, Overgaard M, Nielsen HM, Overgaard J, Danish Breast Cancer Cooperative Group. Estrogen receptor, progesterone receptor, HER-2, and response to postmastectomy radiotherapy in high-risk breast cancer: the Danish Breast Cancer Cooperative Group. J Clin Oncol. 2008;26:1419-1426. 10.1200/JCO.2007.14.5565 18285604

[73] Agrawal A. Oncoplastic —breast surgery and radiotherapy—Adverse aesthetic outcomes, proposed classification of aesthetic components, and causality attribution. Breast J. 2019;25:207-218. 10.1111/tbj.13193 30710399

[74] Charlson ME , Pompei P, Ales KL, MacKenzie CR. A new method of classifying prognostic comorbidity in longitudinal studies: development and validation. J Chronic Dis. 1987;40:373-383. 10.1016/0021-9681(87)90171-8 3558716

[75] National Audit of Breast Cancer in Older Patients (NABCOP). NABCOP 2022 Annual Report. NABCOP. 2022. Accessed November 2023. https://www.nabcop.org.uk/reports/nabcop-2022-annual-report/

[76] Age gap decision tool. Accessed November 2023. https://AgegapShefAcUk/n.d.

