# Supplementary material for: Treatment strategies for triple-negative primary breast cancer in older women: a systematic review
Source: JNCI Cancer Spectr. 2025 May 15;9(3):pkaf049. doi: 10.1093/jncics/pkaf049 (PMC12199755; doi:10.1093/jncics/pkaf049)
Supplement: pkaf049_Supplementary_Data [file pkaf049_supplementary_data.docx]

**Treatment Strategies for Triple-Negative Primary Breast Cancer in Older Women: A Systematic Review**

**Appendix**

**Appendix A**

The search consisted of terms such as: (“Aged” OR “elderly”, “septuagenarian*” OR “ octogenarian*” OR “nonagenarian” OR “centenarian*” ) AND ( “TNBC” OR “ER negative breast cancer*” OR “Triple Negative Breast Neoplasm* “ ) AND (”survival rate” OR “Disease Free Survival” OR “survival” OR “Treatment Outcome*” OR “treatment*” OR “therapy” OR “cancer specific survival” OR “cancer specific outcome” OR “Progression Free Survival” OR “Remission “OR “recover*”).

**Appendix B**

| **Author** | **Bias Due to Confounding** | **Bias in**  **Selection of**  **Participants** | **Bias in Classification of**  **Interventions** | **Bias due to deviations from intended intervention** | **Bias Due to Missing Data** | **Bias in**  **Measurement of Outcomes** | **Bias in**  **Selection of**  **Reported**  **Result** |
| --- | --- | --- | --- | --- | --- | --- | --- |
| **Xu et al, 2022** | Moderate | Moderate | Low | Low | Low | Low | Moderate |
| **Drapalik et al,**  **2022** | Serious | Moderate | Low | Low | Low | Low | Moderate |
| **Mburu W et al, 2022** | Moderate | Moderate | Low | Low | Low | Low | Moderate |
| **Tang Z et al,**  **2022** | Serious | Moderate | Low | Low | Low | Low | Moderate |
| **Saifi O et al,**  **2022** | Serious | Moderate | Low | Low | Low | Low | Moderate |
| **Janeva et al,**  **2020** | Moderate | Moderate | Low | Low | Low | Low | Moderate |
| **Kaplan HG et al, 2017** | Moderate | Moderate | Low | Low | Low | Low | Moderate |
| **Algan O, et al,**  **2016** | Moderate | Moderate | Low | Low | Low | Low | Moderate |
| **Bulut N et al,**  **2015** | Moderate | Moderate | Moderate | Low | Low | Low | Moderate |
| **Zhu W. et al, 2015** | Serious | Moderate | Low | Low | Low | Low | Moderate |
| **Daisy L. Spoer et al, 2023** | Serious | Moderate | Low | Low | Low | Low | Low |
| **Valachis A et al, 2021** | Moderate | Moderate | Low | Low | Low | Low | Moderate |
| **Haque et al, 2019** | Moderate | Moderate | Low | Low | Low | Low | Moderate |
| **JD Qiu et al,**  **2016** | Moderate | Moderate | Low | Low | Low | Low | Moderate |
| **Kozak et al, 2019** | Moderate | Moderate | Low | Low | Low | Low | Moderate |
| **Tzikas et al,**  **2020** | Moderate | Moderate | Moderate | Low | Low | Low | Moderate |
| **Guo et al, 2023** | Serious | Moderate | Low | Low | Moderate | Low | Low |
| **M Xiu et al,**  **2022** | Moderate | Moderate | Moderate | Low | Moderate | Moderate | Low |
| **Haque et al, 2017** | Moderate | Low | Moderate | Low | Low | Low | Low |
| **Crozier et al,**  **2020** | Moderate | Moderate | Low | Low | Moderate | Low | Moderate |
| **Bhoo-Pathy et al, 2014** | Moderate | Moderate | Low | Low | Low | Low | Low |

1

| **Tan H et al,**  **2023** | Moderate | Low | Low | Low | Low | Low | Low |
| --- | --- | --- | --- | --- | --- | --- | --- |
| **Brown et al,**  **2022** | Moderate | Moderate | Low | Low | Low | Low | Moderate |
| **Zhong et al,**  **2020** | Moderate | Moderate | Moderate | Low | Low | Moderate | Low |
| **Syed BM et al, 2014** | Moderate | Moderate | Low | Low | Low | Low | Moderate |
| **Roy et al, 2024** | Moderate | Low | Low | Low | Low | Low | Low |
| **Honma et al, 2021** | Moderate | Moderate | Low | Low | Low | Low | Moderate |
| **Zhai et al,**  **2020** | Moderate | Low | Moderate | Low | Low | Low | Moderate |
| **Schreiber et al, 2021** | Moderate | Moderate | Low | Low | Low | Low | Moderate |
| **Roy et al, 2023** | Moderate | Low | Moderate | Low | Low | Low | Moderate |

**Table 1B**

| **Author, Year, Country** | **Study type** | **Data source, Country** | **Study period** | **Patients meeting our study criteria (n), age** | **Patient and Tumour Characteristics** | **Treatment Regimen investigated** | **Findings** | **Investigators Recommended Outcomes** | **Knowledge Gaps Identified/Missing** |
| --- | --- | --- | --- | --- | --- | --- | --- | --- | --- |
| Mburu W et al 2022, US | RCS+ IPTW analysis | SEER - US | 2010-2015 | 4598, >66 | - 94% had Mx or breast-conserving surgery.  -Of these, 49% had BCS + RT, 28% had Mx, 14% had Mx + RT, and 9% had BCS. | BCS; BCS plus RT; Mx; and Mx plus RT. | -owTNBC have higher death hazard.  -BCS + RT lowers death incidence.  -5-year cumulative death incidences: BCS + RT (8·35%), Mx (18·56%), Mx + RT (36·02%), BCS (20·44%).  -BCS + RT has lower death hazard.  -Chemotherapy adjustment doesn't change findings.  -BCS + RT improves OS, BCSS (stages I-II). | - Omission of RT after BCS is problematic and needs urgent attention.  - BCS + RT is associated with better prognosis in early-stage TNBC. | -Stage III analysis had lower power.  -Database limits assessing recurrence, margin, family history, nonclinical factors. |
| Saifi O et al 2022, US | RCS+PSM. | SEER - US | 2010-2015 | 5743, >60 | T1-2N0M0. TNBC as first malignancy | BCS vs Mx in early stage TNBC | -BCS + RT improves 5-year OS, BCSS (p<0·001).  - patients >60 years old : better survival with BCS + RT.  -In chemotherapy-received patients, BCS + RT has higher 5-year OS (p = 0·007) and BCSS (p = 0·021) than Mx. | BCS + RT is associated with better OS and BCSS compared to Mx in T1-2N0M0 TNBC patients. | - Retrospective, non-randomized study with limitations.  -SEER lacks locoregional recurrence data.  -HER-2 status data limited to 2010.  -Missing data on BRCA mutation and chemotherapy sequence. |
| Zhong et al 2020, China | RCS | Peking Union Medical College Hospital (PUMCH) database - China | 2010-2016 | 450, ≥70 | Patients were either treated with BCS alone or Mx+ALND | BCS without ALND, SLND, or RT. | -BCS alone and Mx+ALND have similar 5-year DFS.  -No OS, BCSS difference in TNBC. | -BCS is suitable for owTNBC  - Prognosis may depend on tumour characteristics, supporting BCS recommendation for owTNBC. | -Selective bias between BCS, Mx+ALND group, with BCS: more endocrine therapy; Mx+ALND: more chemotherapy.  -TNBC subset has limited sample size. |

Retrospective cohort study (RCS), Inverse probability treatment weighting (IPTW), Propensity score matching (PSM), Breast conserving surgery (BCS), Radiotherapy (RT), Mastectomy (Mx), Axillary lymph node dissection (ALND), sentinel lymph node dissection (SLND), Overall survival (OS), Breast cancer specific survival (BCSS), Triple negative breast cancer (TNBC), older women with Triple negative breast cancer (owTNBC)

**Table 2B**

| **Author, Year, Country** | **Study type** | **Data source, Country** | **Study period** | **Patients meeting our study criteria (n), age** | **Patient and Tumour Characteristics** | **Treatment Regimen investigated** | **Findings** | **Investigators Recommended Outcomes** | **Knowledge Gaps Identified/Missing** |
| --- | --- | --- | --- | --- | --- | --- | --- | --- | --- |
| Xu et al. 2022, US | RCS+ PSM + nomogram. | SEER - US | 2010-2015 | 4465 ≥ 65 | T1–2N0–1 tumours | RT after BCS | -owTNBC favour RT over chemotherapy.  -≥70: poorer OS, BCSS (P<0·001).  -Adjuvant chemotherapy improves OS, but no BCSS change.  -RT improves OS, BCSS in ≥70. | -RT benefits owTNBC, except low-risk subgroup; high-risk group requires RT. | -Study focused on HR-negative tumours.  -Retrospective limitations: nonrandomization, underreporting, and unknown confounders.  -PSM may not cover all factors. |
| Tang Z et al 2022, China | RCS + nomogram | SEER - US | 2010-2015 | 4761, ≥70 | T1-3N0-1M0  Mostly 1A stage | Effects of adjuvant chemotherapy or postoperative RT on OS and BCSS | -RT improves OS + BCSS (p < 0.0001).  -Chemotherapy boosts 3-, 5-, 7-year OS (p < 0·0001), but no  BCSS benefit.  -No chemoradiotherapy linked to worst survival (p < 0·0001).  -Age, chemotherapy, RT predict OS (p < 0·001). | -Results support chemotherapy and RT in owTNBC, aiding management. | -Retrospective study introduces selection bias.  -SEER lacks key confounder information.  -Chemotherapy and RT details missing.  -Absence of external cohorts limits reliability. |
| Algan O, et al. 2016, US | RCS | NCDB - US | 1998-2012. | 10079, ≥ 70 | - BCS or Mx for locally advanced T3-4 or N1-3, M0 tumours.  -Excluded metastasis or stage IV disease. | Impact of postoperative RT in TNBC. | -RT improves TNBC OS significantly.  -RT use decreases with increasing age. | There is improvement in OS with the addition of RT after BCS. | -Possible selection bias with radiation.  -RT more likely in healthier patients.  -NCDB limitations, limited treatment outcome information. |
| Haque et al. 2019, US | RCS+ PSM | NCDB - US | 2004-2014 | 8526, ≥70 | T1‐2N0M0 TNBC treated with BCS.  74% received postoperative RT | Omission of post BCS in owTNBC. | -RT use decreases with age.  -Adjuvant RT boosts 5-year OS (P < 0·001).  -Omission of RT linked to poorer OS (P < 0·001).  -No RT cohort has higher Mx rate, diminishes with age. | caution must be exercised when considering omission of adjuvant RT in node‐negative TNBC patients. | -Retrospective studies may exhibit patient biases.  -Aggressive interventions linked to favourable disease.  -5-year OS difference observed, mitigated by stratification.  -NCDB codes chemotherapy but not specific types.  -Lack of information on LR, CSS, RT. |
| Haque et al 2017, US | RCS | SEER - US | 2010-2013 | 788, ≥70 | T1N0M0 treated with BCS. | Postoperative RT utilisation in older breast cancers. | -Adjuvant RT improves BCSS in ER-/HER2-.  -Poor differentiation, ER-/HER2- favour RT; predict poorer outcomes. | - age should not be a consideration to change management in TNBC. | -SEER lacks key prognostic factors.  -Retrospective nature limits follow-up time.  -BCSS differences, short follow-up time.  -SEER lacks systemic agent information.  -Miscoding potential in SEER database.  -Data limited to 2010-2013.  -Lack of focus on TNBC. |
| Crozier et al 2020, US | RCS + PSM | NCDB - US | 2004-2014 | 16,062 ≥ 70 | Primary TN tumour ≥ 6mm or ≥ one positive lymph node. Excluded non-invasive, T1aN0M0, M1. | Addition of chemotherapy to local therapy on OS in owTNBC. | -Chemotherapy improves OS (p<0·0001), persisting after adjustment for node status and comorbidity.  -RT improves overall survival (p<0·0001).  -No clear age limit for chemotherapy benefit. | -Data supports considering chemo and RT in treating women aged 70+ with TNBC. | -PSM balanced treatment groups.  -Data limitations: geography, facility type captured.  -Heterogeneous ageing, complex to assess.  - retrospective design, registry-based.  -Missing HER2 data excluded patients.  -Limited data on functional status, cognition. |
| Bhoo-Pathy et al 2014, Malaysia | RCS | 5 Institutional databases across Asia - UMMC, Malaysia; NCCS, Singapore; NUH, Singapore, ; TTSH, Singapore; The University of Hong Kong; QMTWH, Hong Kong. | 2006-2011 | 1138, ≥ 65 | - Stage IV or BCS without postoperative RT excluded. | Comparing Survival between patients receiving Mx only, BCS and Mx with RT | -Age modifies locoregional treatment impact on survival.  -Older age: No survival advantage with BCT, post-Mx RT. | Age modifies the association between adjuvant RT and survival in TNBC. | -Socioeconomic status mismatch may impact survival.  -Potential overestimation due to socioeconomic differences.  -Selection bias in observational study.  -Post-Mx RT given based on indication.  -Adjustments made, but unmeasured confounders remain. |
| Zhai et al 2020, China | RCS | SEER - US | 2010-2015 | 1715, ≥ 70 | -T1N0M0 TNBC females post-surgery.  - node-negative status. | Effects of adjuvant chemotherapy or postoperative RT on OS and BCSS T1N0 M0 TNBC . | -owTNBC favour RT over chemotherapy.  -≥70: poorer OS, BCSS (P<0·001).  -Adjuvant chemotherapy improves OS, no significant BCSS change.  -RT improves OS, BCSS in ≥70. | -Consider RT for postoperative TNBC patients aged 70+ with T1N0M0 TNBC based on the evaluation of health status. | -Unable to assess comorbidities and neoadjuvant therapies.  -Convergence issue in stratified multivariable analysis.  -Unable to address potential immortal time bias. |

Retrospective cohort study (RCS), Propensity score matching (PSM), Radiotherapy (RT), Breast conserving surgery (BCS), Overall survival (OS), Breast cancer specific survival (BCSS), Triple negative breast cancer (TNBC), Older women with triple negative breast cancer (owTNBC), mastectomy (Mx), oestrogen receptor (ER).

**Table 3B**

| **Author, Year, Country** | **Study type** | **Data source, Country** | **Study period** | **Patients meeting our study criteria (n), age** | **Patient and Tumour Characteristics** | **Treatment Regimen investigated** | **Findings** | **Investigators Recommended Outcomes** | **Knowledge Gaps Identified/Missing** |
| --- | --- | --- | --- | --- | --- | --- | --- | --- | --- |
| Tang Z et al 2022, China | RCS+ nomogram | SEER - US | 2010-2015 | 4761, ≥70 | limited to T1-3N0-1M0.  49% at IA stage | Impact of RT or chemotherapy after surgery on long-term OS and CSS. | -Radiation benefits OS, CSS (p < 0·0001).  -Chemotherapy boosts 3-, 5-, 7-year OS (p < 0·0001), limited CSS benefit (p = 0·17).  -No chemoradiotherapy worsens OS, CSS (p < 0·0001).  -Age, chemotherapy, RT predict OS (p < 0·001).  -Nomogram validated, C-index 0.716 (1429 cases). | -Results support active TNBC management in older patients. | -Retrospective study prone to selection bias.  -Missing data on Ki-67, 21-GRS, comorbidities.  -Adjuvant chemo and RT details unavailable.  -Nomogram reliability limited by lack of cohorts. |
| Janeva et al 2020, Sweden | RCS + PSM | Swedish National Breast Cancer Register; Swedish Patient Register; Swedish Cause of Death Register - Sweden | 2009-2016 | 1130, ≥70 | All had early TNBC. | Adjuvant chemotherapy | -Adjuvant Chemotherapy improves 5-year BCSS, OS (p < 0·0001), persisting after PSM (p = 0·029).  -Cox regression: 5-year BCSS, OS (p = 0·013, p = 0·0035). | - owTNBC patients benefit from adjuvant chemotherapy in both BCSS and OS | -Limitations include registry-based, retrospective design.  -Charlson-Deyo index used as frailty proxy in absence of accurate information.  -Lack of details on chemotherapy type. |
| Valachis A et al 2021, Sweden | RCS | Breast Cancer Data Base Sweden (BCBaSe) - Sweden | 2007-2012 | 413, ≥70 |  | Chemotherapy treatment patterns, risk factors for hospitalisation and causes of death in owTNBC. | -Age and comorbidity associated with lower chemotherapy use.  -Chemotherapy use links to increased hospitalization risk.  -Higher stage, comorbidity, increase breast cancer mortality.  -CCI ≥ 2 raises non-breast cancer mortality.  -Age, advanced stage and comorbidity independently raise overall mortality. | - need to tailor TNBC treatment for older patients, considering various factors.  -No survival benefit observed with chemotherapy in the study cohort. | -Limited patients in specific subgroups.  -Retrospective study may suffer bias.  -Caution needed in interpreting chemotherapy impact as study was retrospective and not designed to answer this particular question. |
| Guo et al 2023, China | RCS + IPTW | SEER - US | 2010-2018 | 4610, ≥70 | ductal TNBC treated with surgery; survival ≥ 1 month.  Patients treated with BCS without RT excluded. | Effects of adjuvant chemotherapy | -Chemotherapy yields better 5-year BCSS, OS (p < 0·001).  -IPTW-adjusted Cox regression supports adjuvant chemotherapy (p < 0·001).  -T1ab patients gain no chemotherapy benefit (p > 0·050).  -In >80 age group, OS improves. | - owTNBC patients benefit from adjuvant chemotherapy in survival.  -Caution needed in de-escalated treatment for T1ab patients.  -Consider physical status for chemotherapy recommendations in owTNBC, particularly in >80 groups | -IPTW controlled baseline differences in groups.  -Unavailable factors: Ki67, Charlson–Deyo index, BRCA1/2.  -Regimen changes during 2010–2018.  -Selection bias inevitable in retrospective study. |
| M Xiu et al 2022, China | RCS | Single institutional database - China | 2010-2016 | 177, ≥ 65 | Patients excluded: recurrent/metastatic cancer, non-invasive or bilateral breast cancer, other malignancy, no surgery, T1aN0M0. | current chemotherapy options in owTNBC | - ≥6 chemotherapy cycles improve RFS (P=0·027).  -Chemotherapy (≥1 cycle) boosts BCSS, OS (P=0·046, P=0·029).  -Regimen options differ by age, stage.  -ATAX for 65-69, stage II-III.  -≥70: carboplatin-based, often TCb.  -No RFS difference among regimens. | - owTNBC showed improved BCSS and OS with chemotherapy, suggesting a need for individualised regimes.  -TCb regimen may be feasible when intolerant to anthracycline. | -Longer 59-month follow-up than comparable studies.  -Heterogeneity in patient characteristics, chemotherapy regimens.  -Single-institute design, limited population size.  -Absence of individualized Comprehensive Geriatric Assessment (CGA) data. |
| Brown et al 2022, US | RCS + PSM | NCDB - US | 2004-2017 | 12,090, ≥ 70 |  | Effects of NAST in septuagenarians and octogenarians with HER2+or TNBC | -NAST benefits OS in TNBC,  -Surgery omission after NAST, poor OS.  -Careful selection for NAST crucial.  -NAST improves OS in both groups (p<0·001).  -Decline during NAST associated with poorer OS. | -NAST + surgery is effective for both groups.  -Careful selection is crucial for optimizing outcomes in this population. | -NCDB lacks specific report on NAST regimens/toxicity  -Unclear if NAST was part of original plan.  -No data on disease progression/recurrence. |
| Crozier et al 2020, US | RCS + PSM | NCDB - US | 2004-2014 | 16,062 ≥ 70 | Primary TN tumour ≥ 6mm or ≥ one positive lymph node. Excluded non-invasive, T1aN0M0, M1. | Addition of chemotherapy to local therapy on OS in owTNBC. | -Chemotherapy improves OS (p<0·0001), persisting after adjustment for node status and comorbidity.  -RT improves overall survival (p<0·0001).  -No clear age limit for chemotherapy benefit. | -Data supports considering chemo and RT in treating women aged 70+ with TNBC. | -PSM balanced treatment groups.  -Data limitations: geography, facility type captured.  -Heterogeneous ageing, complex to assess.  - retrospective design, registry-based.  -Missing HER2 data excluded patients.  -Limited data on functional status, cognition. |
| Schreiber et al 2021, US | RCS | SEER - US | 2010-2015 | 3348 ≥ 66 | -About half had CCI ≥1.  -Majority had prior cardiac conditions.  -Only a quarter had non-cardiac conditions. | Effects of ATAX vs TAX based chemotherapy regimen on CSS and OS in node-negative owTNBC | -Aged 76+, less likely for chemotherapy (p<0·0001).  -Older women, less likely for ATAX (p<0·0001).  -Adjuvant chemotherapy improves CSS, OS.  -TAX better than ATAX for CSS, OS.  -ATAX doesn't improve CSS in T stages. | Consider use of chemotherapy in older patients who are expected to obtain benefit from TAX, with a pause to the use of ATAX in this population. | -Longer follow-up may reveal more complications.  -Bias: longer life expectancy, chemotherapy receipt.  -Limited to fee-for-service population practices.  -No data on cause of death, toxicity, or quality of life. |
| Roy et al 2023, US | RCS | SEER - US | 2010-2015 | 1106 ≥ 66 | -42.6% had T2 tumours.  -60.6% had CCI score 0. | Evaluate the benefit of adjuvant ATAX vs TAX in node-positive owTNBC. | -Chemotherapy improves 3-year BCSS, OS (P < 0·01).  -ATAX and TAX have similar outcomes (P = 0·80, P = 0·79).  -No significant heart failure difference (P = 0·45).  -Heart failure linked to less ATAX (P = 0·04).  -Older patients benefit from ATAX (P = 0·02).  -Trend towards worse CSS in 1-3 nodes with ATAX (P = 0·05). | - owTNBC less likely for adjuvant chemo; clear survival benefit.  -ATAX lacks survival benefit; but could be beneficial with ≥4 lymph nodes. | -Retrospective data may introduce bias.  -Ignored chemotherapy toxicities and quality-of-life changes.  -Unobservable health differences could influence decisions. |
| Roy et al 2024, US | RCS | SEER - US | 2010-2015 | 2215 ≥ 66 | 42.6% had T2 tumours.  -60.6% had CCI score 0. | incidence of major adverse cardiovascular events (MACE) in TNBC after adjuvant chemotherapy | -Younger patients prefer Anthracycline and Taxane (ATAX) (p<0·01).  -No higher risk in ATAX for:  ---Myocardial infarction (p=0·91).  ---Heart failure (p<0·01).  ---Potentially fatal arrhythmia (p=0·12).  ---Cerebrovascular accident (p=0·20).  -No difference in OS, CSS for cardiac outcomes (TAX vs. ATAX). | age may serve as a predictor for worse survival **once** a cardiac event occurs. | -Retrospective data may introduce bias.  -Ignored chemotherapy toxicities and quality-of-life changes.  -3-year follow-up limits cardiovascular event detection.  -Unobservable health differences may lead to bias.  -SEER registries lack information on risk factors.  -Small sample size hinders definitive conclusions. |
| Zhai et al 2020, China | RCS | SEER - US | 2010-2015 | 1715, ≥ 70 | -T1N0M0 TNBC females post-surgery.  - node-negative status. | Effects of adjuvant chemotherapy or postoperative RT on OS and BCSS T1N0 M0 TNBC . | -Older patients favour RT over chemotherapy.  -≥70: poorer OS, BCSS (P<0·001).  -Adjuvant chemotherapy improves OS, non-significant BCSS change.  -RT improves OS, BCSS in ≥70. | -Consider RT for postoperative TNBC patients aged 70+ with T1N0M0 TNBC based on the evaluation of health status. | -Unable to assess comorbidities and neoadjuvant therapies.  -Convergence issue in stratified multivariable analysis.  -Unable to address potential immortal time bias. |

Retrospective Cohort Study (RCS), Inverse Probability Treatment Weighting (IPTW), Propensity score matching (PSM) Breast conserving surgery (BCS), Radiotherapy (RT), Mastectomy (Mx), Triple negative breast cancer (TNBC), Axillary lymph dissection (ALND), Sentinel lymph dissection (SLD), Overall survival (OS), Breast cancer specific survival (BCSS), Propensity score matching (PSM), anthracycline-and-taxane (ATAX), Taxane alone (TAX), major adverse cardiovascular events (MACE), Androgen Receptor (AR), Tumour infiltrating lymphocytes (TILs)

**Table 4B**

| **Author, Year, Country** | **Study type** | **Data source, Country** | **Study period** | **Patients meeting our study criteria (n), age** | **Patient and Tumour Characteristics** | **Treatment Regimen investigated** | **Findings** | **Investigators Recommended Outcomes** | **Knowledge Gaps Identified/Missing** |
| --- | --- | --- | --- | --- | --- | --- | --- | --- | --- |
| Drapalik et al. 2022, US | RCS | NCDB - US | 2005-2017 | 36042, ≥70 | unilateral, invasive TNBC | Disparities in treatment and outcomes based on age | -Adjuvant chemotherapy, NAC improve survival (P < 0·0001).  -Partial Mx common in 41-70, >70.  -Unilateral Mx more in >70.  -Poorer survival with unilateral Mx, no surgery (P < 0·0001).  -Radiation improves survival in all ages (P < 0·05).  -In oldest, omission of chemotherapy, axillary surgery, radiation.  -Age independent predictor of treatment type. | Multimodal therapy options should be considered for all patients, irrespective of age. | -NCDB lacks locoregional recurrence, DFS data.  -Missing details: provider recommendations, patient preferences.  -Physiologic age, chemotherapy regimens, BRCA status not captured. |
| Kaplan HG et al 2017, US | RCS | internal institution breast cancer registry - US | 1990-2014 | 159, ≥65 | Primary tumours, stage I-III.  84% of cohort was of white race. | Surgery, Chemotherapy and RT treatment outcomes in owTNBC. | -≥75: Surgery, radiation common (48%).  -≥75: More likely to have Mx.  -Radiation similar across age groups.  -≥75: Less likely doxorubicin regimen (P<0·001).  -Chemotherapy completion declines with age (P=0·001).  -Age not significant predictor in Cox model. | -In TNBC patients aged 75+, lower treatment levels yield outcomes similar to younger patients, indicating comparable effectiveness across age groups. | -HER-2 testing started in 1997, retrospectively for ER/PR-negative patients diagnosed from 1990 to 1996. |
| Bulut N et al. 2015 Turkey | RCS | Single Institution database - Turkey | 2008-2013 | 58, ≥60 | . | histopathologic features and survival of older vs younger TNBC. | -Chemotherapy benefits both age groups (DFS, OS).  -More comorbid diseases in older patients (p=0·001).  -Adjuvant RT differs between age groups (p=0·001).  -No diminishing chemotherapy benefit with age.  -No difference in DFS, OS (p=0·914, p=0·939) | -Similar survival in older and younger TNBC patients with neoadjuvant and adjuvant chemotherapy and adjuvant RT suggests common tumour characteristics. | -Retrospective study poses selection bias risk.  -Variable chemotherapy regimens were administered. |
| Zhu W. et al. 2015, China | RCS | SEER - US | 2010-2011 | 2207, ≥ 70 | Female primary non-metastatic TNBC.  Stage IV tumours and and patients with other malignancies were excluded. | early BCSS and OS patterns (within 2 years of diagnosis) in older and younger TNBC. | - >70 Predicts poor CSS, OS (p<0·001).  -owTNBC: Benign biological features (p<0·001), less lymph node metastasis (p<0·001), earlier TNM stage (p<0·001), better differentiation (p<0·001).  -Underuse of RT more common in owTNBC with stage II or III diseases (p<0·001).  -owTNBC patients undergoing surgery had better immediate CSS (p<0·001).  -Underuse of curative treatment, especially RT, more common in older women with stage II or III diseases.  -Surgery alone: Age-related survival gap (p<0·001).  -TNBC cohort with surgery and RT: Age-caused gap narrowed (p=0·126). | Denying SoC anti-cancer treatment to this cohort merely based on age is not fully justified. | -SEER lacks key comorbidity and chemotherapy data.  -SEER database doesn't explain age-related treatment differences.  -Subgroup analysis has limited statistical power. |
| Daisy L. Spoer et al. 2023, US | RCS | Three institutions, US | 2018-2022 | 21, ≥80 |  | Differences in outcomes based on level of care in owTNBC | -No significant mortality differences between SoC and insufficient care groups.  -Age, T/N grade, TNM stage not significantly associated with deferral.  -Treatment deferral linked to poorer functional status; ECOG III significantly associated with deferral (P=0·0140). | This pilot study emphasizes that SoC treatment offerings should be directed by tumour biology and individual patient profiles rather than age alone. | -Limited cases hindered comprehensive treatment analysis.  -Excluded non-surgical patients, introducing potential bias.  -Lack of separation by chemotherapy regimens and RT details. |
| JD Qiu et al. 2016, China | RCS | Single Institutional database - China | 2004-2008 | 66, ≥60 years |  | investigate the clinicopathological features, recurrence, metastasis, treatment methods and prognosis of the younger versus owTNBC | -Older group had higher survival.  -Older group received less RT, chemotherapy.  -Significant differences in RT, chemotherapy (P < 0·001).  -OwTNBC patients more often received CMF (P < 0·001). | - The older and younger TNBC patients may belong to different biological subtypes. - More conservative and cautious attitude in choosing the post operative adjuvant treatment for owTNBC. | -Retrospective study may have selection bias.  -Small sample size hinders statistical power.  -Conflicting data on DFS  -Lack of specified adjuvant RT details. |
| Kozak et al 2019, US | RCS | SEER - US | 2010-2014 | 4221 ≥70 |  | Postoperative RT or adjuvant Chemotherapy in women with TNBC. | -Women ≥70 received less chemotherapy, radiation.  -No BCSM difference in adjuvant treatment.  -BCSM difference diminished with both chemotherapy and RT.  -Age not a significant BCSM predictor.  -OwTNBC equally chose conservation, Mx. | owTNBC benefit from more aggressive therapy, and that undertreatment, rather than age itself, is in part responsible for worse outcomes in this patient population. | -SEER lacks details on chemotherapy, RT.  -Retrospective nature introduces potential bias.  -Reasons for undertreatment not assessable in SEER.  -Adequate follow-up for high-risk population. |
| Tzikas et al 2020, Sweden | RCS | The Swedish regional breast cancer registry - Sweden | 2007-2015 | 96, >74 | Most were ductal invasive carcinomas, grade 3 (80%). | biology, recurrence rate, metastatic patterns and survival times in TNBC between older and young patients. | -Adjusting for chemotherapy, survival differences vanished.  -owTNBC favoured Mx, less axillary surgery.  -Younger patients received more chemotherapy (p=0·0005).  -Chemotherapy type varied with age (p=0·004).  -owTNBC received less radiation therapy. | owTNBC patients in good general condition could benefit from adjuvant chemotherapy and that less toxic regimens can be an efficient option. | -Long follow-up, 10/524 lost, robust results.  -Small patient number, notably in metastatic comparison.  -Missing BRCA, gene profiling, comorbidity data. |
| Tan H et al 2023, China | RCS | SEER - US | 2011-2014 | 2610, ≥75 |  | Age as a prognostic factor in TNBC | -owTNBC OS and BCSS significantly lower.  -Multivariate analysis identified independent TNBC risk factors.  -owTNBC patients had lower surgery.  -Excluding treatments increased hazard ratio.  -Poor prognosis partly due to treatment disparities.  - Age is an independent risk factor for the prognosis of TNBC patients. | Older patients receive less active treatment than younger counterparts. | -Small ≥75 cohort, study limitations overlooked.  -Inherent selection bias in retrospective study.  -SEER lacks key prognostic factors recording.  -No direct intervention, lacks systemic agent details.  -Possibility of miscoded treatments, large sample.  -Data predates 2015 PRIME II study. |
| Syed BM et al 2014, UK | RCS | Single Institutional database (older patients). Younger patients from previously characterised institutional database (Nottingham/Tenovus series) - UK | 1973-2010 | 127, ≥70 | owTNBC had significantly lower expression of Ki67, more normal p53 and higher expression of Bcl2 than younger women. | analyse the biology and clinical outcome of younger vs owTNBC. | -Fewer older patients received chemotherapy.  -owTNBC less aggressive.  -No significant age-related clinical differences.  -Similar outcomes irrespective of age. | Biological differences in the tumours could explain why older women did not have worse outcomes despite less treatment. | -TNBC management changed, minimal impact.  -Series compared at five-year follow-up.  -Retrospective study limitations include sample size, completeness, confounding, miscoding, selection bias. |
| Honma et al 2021, Japan | RCS | Toho University Medical Center Omori Hospital (Tokyo, Japan), Aichi Cancer Center (Nagoya, Japan), Tokyo Metropolitan Geriatric Hospital (Tokyo, Japan), Yokohama City University Hospital (Yokohama, Japan), and Kagawa University Hospital (Kagawa, Japan) - Japan | 2004-2013 | 75, ≥ 75 | stage IV, bilateral tumours or patients with NAST have been excluded. | clinicopathological characteristics of younger vs owTNBC. | -Older patients received less adjuvant therapy.  -Older group: Higher AR, lower CK5/6, TILs.  -AR positivity benefits older patients.  -TIL status not influential in outcomes.  -AR-positive TNBC may avoid chemotherapy.  -No age-based differences in histology.  -Similar outcomes across histological types. | AR positivity was a predictor of decreased recurrence in older patients. AR-positive tumours, but not tumours with high levels of TILs, were associated with a favourable clinical outcome in older patients, suggesting the importance of examining AR. | -Neoadjuvant therapy rarely used in older patients.  -Small study size for pStage III  -Adjuvant therapy less frequent in owTNBC. |

Retrospective Cohort Study (RCS). Breast cancer specific survival (BCSS), Radiotherapy (RT), Older women with triple negative breast cancer (owTNBC), Triple negative breast cancer (TNBC), Overall survival (OS), Neoadjuvant chemotherapy (NAC), Mastectomy (Mx), Disease free survival (DFS), Tumour Node Metastasis (TNM), Standard of Care (SoC), Disease free survival (DFS), Androgen Receptor (AR), Tumour infiltrating lymphocytes (TILs), Multidisciplinary team (MDT),
